# Supplementary material for: All-cause mortality among patients treated with repurposed antivirals and antibiotics for COVID-19 in Mexico City: A real-world observational study
Source: EXCLI J. 2021 Feb 4;20:199–222. doi: 10.17179/excli2021-3413 (PMC7898041; doi:10.17179/excli2021-3413)
Supplement: Supplementary appendix [file EXCLI-20-199-s-001.pdf]

SALUD Secretaría de Salud | IMSS | ISSSTE | PEMEX | SEDENA Secretaría de la Defensa Nacional | SEMAR Secretaría de Marina | SNDIF Sistema Nacional DIF | INPI Instituto Nacional de los Pueblos Indígenas

Epidemiologic study of suspected viral respiratory disease

GENERAL INFORMATION

Name of medical unit: ______

Date of registration in the platform: ______ dd/mm/yyyy Platform number: ______

Last Name: ______ Second Last Name: ______ Name(s): ______

Date of birth: Day: ______ Month: ______ Year: ______ CURP: ______

Sex: Man: ______ Woman: ______

Pregnant patient: Yes ______ No ______ Months of pregnancy: ______

Patient in puerperium Yes ______ No ______ Days of puerperium ______

Nationality: Mexican: ______ Foreigner: ______ Migrant: Yes ______ No ______ Country of nationality: ______ Country of origin: ______

Visited countries in the last three months: 1 ______ 2 ______ 3 ______ Other: ______ Date of entry into Mexico: ______

Country of birth: ______ Place of birth (state): ______

Place of residency (state): ______ Municipality of residence: ______

Locality: ______

Street: ______ Number: ______

Between streets: ______ & ______

Suburb: ______ PC: ______ Telephone: ______

Indigenous self-identification: Yes ______ No ______ Speaker of indigenous language: Yes ______ No ______

Occupation: ______

Is the patient affiliated to an educational institution? ______

CLINICAL DATA

Admitted to (service): ______

Type of attention: 1=Ambulatory ______ 2=Hospitalization ______

Date of admission: ______ dd/mm/yyyy Date of symptom onset: ______ dd/mm/yyyy

Since the onset of symptoms, has the patient…

…had any of the following signs or symptoms?

	Yes	No
Sudden onset of symptoms		
Fever		
Cough		
Headache		
Shortness of breath		
Irritability		
Chest pain		
Chills		
Sore throat		
Myalgias		
Arthralgias		
Anosmia		
Dysgeusia		
Rhinorrhea		
Conjunctivitis		

Comorbidities

	Yes	No
Diabetes		
COPD		
Asthma		
Immunosuppression		
Hypertension		
HIV/AIDS		
Cardiovascular disease		
Obesity		
Chronic kidney disease		
Smoker		
Other		

Specify any others: ______

Other symptoms	Yes	No
Abrupt deterioration		
Diarrhea		
Polypnea		
Abdominal pain		
Vomit		
Cyanosis		

Probable diagnosis: 1=Influenza-like illness (ILI)* ______ 2=Severe acute respiratory infection (SARI) ______

*ILI is a mild respiratory disease

TREATMENT

Since the onset of symptoms, has the patient…

…been treated with antipyretics? Yes / No

…been treated with antivirals? Yes / No

If the answer was yes:

Specify the antiviral: ______ 1=Amantadine 2=Rimantadine 3=Oseltamivir 4=Zanamivir 5=Other, specify: ______

When was the antiviral medication started? ______ dd/mm/yyyy

In the medical unit…

…were antibiotics started? Yes / No

…were antivirals started? Yes / No

Specify the antiviral: ______ 1=Amantadine 2=Rimantadine 3=Oseltamivir 4=Zanamivir 5=Other, specify: ______

EPIDEMIOLOGICAL HISTORY

Has the patient had any contact with persons with respiratory disease in the last two weeks? Yes / No

During the weeks prior to symptom onset did the patient have contact with:

	Yes	No
Birds:		
Swine:		

Other animals: ______

Recent travels in the 7-day period before the onset of signs/symptoms: Yes / No

Country: ______ City: ______

Did the patient receive last-year influenza vaccination? Yes / No

Date of vaccination: ______ dd/mm/yyyy

LABORATORY

Was a sample for testing obtained? Yes / No

Laboratory that will be processing the sample: ______

Site of sample: ______ 1=Pharyngeal swab 2=Nasopharyngeal swab 3=Bronchoalveolar lavage 4=Pulmonary biopsy

Date sample was obtained: ______ dd/mm/yyyy

Result: ______

FOLLOW-UP

Follow-up status: ______ 1=Discharged 2=Undergoing treatment/Referred/Domiciliary follow-up/End of follow-up 3=Severe case 4=Mild case 5=Death*

In case of discharge: Indicate cause of discharge: ______ 1=Improvement 2=Curation 3=Voluntary 4=Transferred

Has the patient been admitted to an ICU at any moment of the disease? Yes / No

Has the patient been put on invasive mechanical ventilation at any moment of the disease? Yes / No

Has the patient been diagnosed with pneumonia at any moment of the disease? Yes / No

Date of discharge: ______ dd/mm/ yyyy

Specify if this is a positive COVID-19 case by association or clinical-epidemiological ruling: *

** Mark with an X only one of the following options*

a. Confirmed COVID-19 by clinical-epidemiological association ______

b. Confirmed COVID-19 by clinical-epidemiological ruling (applies only for deaths) ______

c. No (none of the previous options) ______

Death: Date of death: ______ dd/mm/yyyy

Death Certificate (number): ______ ***Death due to influenza or COVID-19** Yes / No

*Attach a copy of the death certificate if the patient met criteria for suspected viral respiratory disease case

Name and position of person registering data

______

______

Name and position of authorizing person

______

______

Date of elaboration: ______ dd/mm/yyyy
