# Supplementary material for: All-cause mortality among patients treated with repurposed antivirals and antibiotics for COVID-19 in Mexico City: A real-world observational study
Source: EXCLI J. 2021 Feb 4;20:199–222. doi: 10.17179/excli2021-3413 (PMC7898041; doi:10.17179/excli2021-3413)
Supplement: Supplementary information [file EXCLI-20-199-s-002.pdf]

EXCLI Journal 2021;20:199-222 – ISSN 1611-2156
Received: January 19, 2021, accepted: February 01, 2021, published: February 04, 2021

Supplementary material to:

Original article:

ALL-CAUSE MORTALITY AMONG PATIENTS TREATED WITH REPURPOSED ANTIVIRALS AND ANTIBIOTICS FOR COVID-19 IN MEXICO CITY: A REAL-WORLD OBSERVATIONAL STUDY

Javier Mancilla-Galindo[1,2], Jorge Óscar García-Méndez[2,3,4], Jessica Márquez-Sánchez[5], Rodrigo Estefano Reyes-Casarrubias[2,3], Eduardo Aguirre-Aguilar[6], Héctor Isaac Rocha-González[7], Ashuin Kammar-García[6,7,*]

1 Unidad de Investigación UNAM-INC, Instituto Nacional de Cardiología Ignacio Chávez, Mexico City, Mexico
2 Facultad de Medicina, Universidad Nacional Autónoma de México, Mexico City, Mexico
3 Departamento de Posgrado y Educación Médica Continua, Instituto Nacional de Cancerología, Mexico City, Mexico
4 Departamento de Infectología, Fundación Clínica Médica Sur, Mexico City, Mexico
5 Departamento de Infectología, Instituto Nacional de Pediatría, Mexico City, Mexico
6 Departamento de Atención Institucional Continua y Urgencias, Instituto Nacional de Ciencias Médicas y Nutrición Salvador Zubirán, Mexico City, Mexico
7 Sección de Estudios de Posgrado e Investigación, Escuela Superior de Medicina, Instituto Politécnico Nacional, Mexico City, Mexico

* **Corresponding author:** A. Kammar-García, Departamento de Atención Institucional Continua y Urgencias, Instituto Nacional de Ciencias Médicas y Nutrición Salvador Zubirán, Mexico City, Mexico, Vasco de Quiroga 15, Tlalpan, Col. Belisario Domínguez Sección XVI, CP 14080 Ciudad de México, México. Tel: +52 5554870900, ext. 5010. E-mail: kammar_nutrition@hotmail.com

https://orcid.org/0000-0002-3875-0945 (Ashuin Kammar-García)

http://dx.doi.org/10.17179/excli2021-3413

This is an Open Access article distributed under the terms of the Creative Commons Attribution License (http://creativecommons.org/licenses/by/4.0/).

EXCLI Journal 2021;20:199-222 – ISSN 1611-2156
Received: January 19, 2021, accepted: February 01, 2021, published: February 04, 2021

Supplementary Table 1: Signs and symptoms in ambulatory patients

Supplementary Table 2: Signs and symptoms in hospitalized patients

Supplementary Table 3: Baseline and follow-up characteristics of survivors and non-survivors

Supplementary Table 4: Survival analysis of patients undergoing IMV

Supplementary Table 5: Survival analysis of patients admitted to ICU

Supplementary Table 6: Survival analysis of non-pregnant/puerperal adults

Supplementary Table 7: Survival analysis of children and adolescents

Supplementary Table 8: Survival analysis of pregnant women

Supplementary Table 9: Unadjusted Cox regression models for mortality in all patients, ambulatory, hospitalized, non-critical, and critical patients

Supplementary Table 10: Mortality risk in patients undergoing IMV

Supplementary Table 11: Mortality risk in patients admitted to ICU

Supplementary Table 12: Mortality risk in non-pregnant/puerperal adults

Supplementary Table 13: Mortality risk in children and adolescents

Supplementary Table 14: Mortality risk in pregnant women

Supplementary Table 15: E-values for the general population, ambulatory, hospitalized, non-critical, and critical patients

Supplementary Table 16: E-values for IMV, ICU, Non-pregnant/puerperal adults, and children and adolescents

Supplementary Table 17: Baseline characteristics of propensity score-matched patients treated with antivirals and controls

Supplementary Table 18: Baseline characteristics of propensity score-matched patients treated with Oseltamivir and controls

Supplementary Table 19: Baseline characteristics of propensity score-matched patients treated with antibiotics and controls

Supplementary Table 20: Baseline characteristics of patients with complete dates of antiviral initiation

Supplementary Table 21: Survival analysis of early and late use of antivirals

EXCLI Journal 2021;20:199-222 – ISSN 1611-2156
Received: January 19, 2021, accepted: February 01, 2021, published: February 04, 2021

Supplementary Figure 1: Density functions of propensity score-matched patients treated with antivirals and controls before and after matching

Supplementary Figure 2: Density functions of propensity score-matched patients treated with Oseltamivir and controls before and after matching

Supplementary Figure 3: Density functions of propensity score-matched patients treated with antibiotics and controls before and after matching

Supplementary Figure 4: Timing of symptom onset, initiation of antiviral, and hospitalization

Supplementary Figure 5: Comparison of hospitalization rates between early and late use of antivirals

Supplementary Appendix: Case form: Epidemiologic study of suspected viral respiratory disease

EXCLI Journal 2021;20:199-222 – ISSN 1611-2156
Received: January 19, 2021, accepted: February 01, 2021, published: February 04, 2021

Supplementary Table 1: Signs and symptoms in laboratory-confirmed COVID-19 ambulatory patients treated with antivirals and/or antibiotics in 603 accredited medical units in Mexico City between February 24, 2020 and September 14, 2020

	No antiviral/ antibiotic n=98060	Acyclovir n=30	Amantadine n=282	Lopinavir-Ritonavir n=8	Oseltamivir n=3187	Rimantadine n=48	Zanamivir n=19	Antibiotic only n=8268	Total n=109902	p value
Number of signs and symptoms, mean (SD)	5.8 (3.8)	6.8 (3.3)[a]	7.2 (3.6)	7.9 (5.3)	7.8 (3.3)[a]	7.6 (3.2)[a]	6.7 (4.6)	7.0 (3.5)[a]	5.9 (3.8)	<0.0001
Fever	52966 (54)	21 (70)	169 (59.9)	5 (62.5)	2465 (77.3)	31 (64.6)	13 (68.4)	5471 (66.2)	61141 (55.6)	<0.0001
Cough	65216 (66.5)	19 (63.3)	216 (76.6)	6 (75)	2583 (81)	34 (70.8)	15 (78.9)	6071 (73.4)	74160 (67.5)	<0.0001
Sore throat	41927 (42.8)	12 (40)	144 (51.1)	3 (37.5)	1611 (50.5)	24 (50)	5 (26.3)	4388 (53.1)	48114 (43.8)	<0.0001
Shortness of breath	19091 (19.5)	11 (36.7)	63 (22.3)	4 (50)	1010 (31.7)	12 (25)	8 (42.1)	2237 (27.1)	22436 (20.4)	<0.0001
Irritability	16364 (16.7)	5 (16.7)	70 (24.8)	2 (25)	720 (22.6)	11 (22.9)	7 (36.8)	1593 (19.3)	18772 (17.1)	<0.0001
Diarrhea	22096 (22.5)	10 (33.3)	86 (30.5)	3 (37.5)	981 (30.8)	12 (25)	7 (36.8)	2456 (29.7)	25651 (23.3)	<0.0001
Chest pain	23150 (23.6)	9 (30)	87 (30.9)	2 (25)	1158 (36.3)	19 (39.6)	7 (36.8)	2494 (30.2)	26926 (24.5)	<0.0001
Chills	32621 (33.3)	14 (46.7)	139 (49.3)	6 (75)	1585 (49.7)	27 (56.3)	5 (26.3)	3482 (42.1)	37879 (34.5)	<0.0001
Headache	67181 (68.5)	21 (70)	202 (71.6)	6 (75)	2575 (80.8)	31 (64.6)	13 (68.4)	6022 (72.8)	76051 (69.2)	<0.0001
Myalgias	47106 (48)	23 (76.7)	175 (62.1)	5 (62.5)	2053 (64.4)	32 (66.7)	8 (42.1)	4993 (60.4)	54395 (49.5)	<0.0001
Arthralgias	42161 (43)	18 (60)	155 (55)	4 (50)	1881 (59)	31 (64.6)	6 (31.6)	4530 (54.8)	48786 (44.4)	<0.0001
Abrupt deterioration	38549 (39.3)	15 (50)	157 (55.7)	5 (62.5)	1923 (60.3)	24 (50)	6 (31.6)	4471 (54.1)	45150 (41.1)	<0.0001
Rhinorrhea	28354 (28.9)	6 (20)	107 (37.9)	4 (50)	1079 (33.9)	23 (47.9)	6 (31.6)	2744 (33.2)	32323 (29.4)	<0.0001
Polypnea	7412 (7.6)	3 (10)	37 (13.1)	3 (37.5)	381 (12)	3 (6.3)	3 (15.8)	859 (10.4)	8701 (7.9)	<0.0001
Vomit	6406 (6.5)	2 (6.7)	18 (6.4)	0 (0)	309 (9.7)	6 (12.5)	1 (5.3)	696 (8.4)	7438 (6.8)	<0.0001
Abdominal pain	11502 (11.7)	3 (10)	49 (17.4)	0 (0)	646 (20.3)	8 (16.7)	4 (21.1)	1065 (12.9)	13277 (12.1)	<0.0001
Conjunctivitis	12784 (13)	4 (13.3)	53 (18.8)	3 (37.5)	475 (14.9)	11 (22.9)	5 (26.3)	1315 (15.9)	14650 (13.3)	<0.0001
Cyanosis	2843 (2.9)	1 (3.3)	9 (3.2)	1 (12.5)	152 (4.8)	6 (12.5)	2 (10.5)	245 (3)	3259 (3)	<0.0001
Sudden onset of symptoms	32348 (33)	7 (23.3)	93 (33)	1 (12.5)	1359 (42.6)	19 (39.6)	7 (36.8)	3067 (37.1)	36901 (33.6)	<0.0001

Data expressed as frequency (%). P values were calculated by X^2 or One-way ANOVA.

a: Statistical difference with respect to No antiviral/antibiotic
SD: Standard deviation

EXCLI Journal 2021;20:199-222 – ISSN 1611-2156
Received: January 19, 2021, accepted: February 01, 2021, published: February 04, 2021

Supplementary Table 2: Signs and symptoms in laboratory-confirmed COVID-19 hospitalized patients treated with antivirals and/or antibiotics in 329 accredited medical units in Mexico City between February 24, 2020 and September 14, 2020

	No antiviral / antibiotic n=16083	Acyclovir n=6	Amantadine n=37	Lopinavir-Ritonavir n=92	Oseltamivir n=5227	Rimantadine n=13	Zanamivir n=20	Antibiotic only n=5475	Total n=26953	p value
Number of signs and symptoms, mean (SD)	7.9 (3.4)	6 (2.6)	7.9 (3.3)	6.6 (2.9)[a]	8.1 (3.4)[a]	8.8 (2.4)	9.4 (2.8)	6.9 (3.2)[a]	7.8 (3.4)	<0.0001
Fever	13045 (81.1)	4 (66.7)	29 (78.4)	76 (82.6)	4498 (86.1)	11 (84.6)	18 (90)	4298 (78.5)	21979 (81.5)	<0.0001
Cough	13151 (81.8)	5 (83.3)	29 (78.4)	61 (66.3)	4472 (85.6)	8 (61.5)	20 (100)	4300 (78.5)	22046 (81.8)	<0.0001
Sore throat	6918 (43)	0 (0)	15 (40.5)	28 (30.4)	2090 (40)	5 (38.5)	17 (85)	1853 (33.8)	10926 (40.5)	<0.0001
Shortness of breath	11970 (74.4)	3 (50)	22 (59.5)	62 (67.4)	4160 (79.6)	10 (76.9)	15 (75)	4264 (77.9)	20506 (76.1)	<0.0001
Irritability	3096 (19.3)	0 (0.0)	11 (29.7)	5 (5.4)	1273 (24.4)	3 (23.1)	6 (3)	932 (17)	5326 (19.8)	<0.0001
Diarrhea	3725 (23.2)	1 (16.7)	10 (27)	16 (17.4)	1199 (22.9)	3 (23.1)	6 (30)	1038 (19)	5998 (22.3)	<0.0001
Chest pain	6374 (39.6)	3 (50.0)	18 (48.6)	20 (21.7)	1821 (34.8)	6 (46.2)	8 (40)	1675 (30.6)	9925 (36.8)	<0.0001
Chills	6784 (42.2)	2 (33.3)	19 (51.4)	28 (30.4)	2031 (38.9)	6 (46.2)	12 (60)	1521 (27.8)	10403 (38.6)	<0.0001
Headache	11712 (72.8)	1 (16.7)	25 (67.6)	53 (57.6)	3773 (72.2)	9 (69.2)	16 (80)	3378 (61.7)	18967 (70.4)	<0.0001
Myalgias	10086 (62.7)	4 (66.7)	26 (70.3)	55 (59.8)	3021 (57.8)	11 (84.6)	16 (80)	3052 (55.7)	16271 (60.4)	<0.0001
Arthralgias	9631 (59.9)	4 (66.7)	24 (64.9)	44 (47.8)	2965 (56.7)	11 (84.6)	17 (85)	2899 (52.9)	15595 (57.9)	<0.0001
Abrupt deterioration	10442 (64.9)	5 (83.3)	23 (62.2)	61 (66.3)	3444 (65.9)	10 (76.9)	14 (70)	3311 (60.5)	17310 (64.2)	<0.0001
Rhinorrhea	3781 (23.5)	0 (0)	11 (29.7)	16 (17.4)	1204 (23)	4 (30.8)	5 (25)	944 (17.2)	5965 (22.1)	<0.0001
Polypnea	4565 (28.4)	0 (0)	9 (24.3)	10 (10.9)	1466 (28)	4 (30.8)	3 (15)	1110 (20.3)	7167 (26.6)	<0.0001
Vomit	1688 (10.5)	1 (16.7)	5 (13.5)	7 (7.6)	561 (10.7)	2 (15.4)	1 (5)	420 (7.7)	2685 (10)	<0.0001
Abdominal pain	2578 (16)	0 (0)	11 (29.7)	8 (8.7)	915 (17.5)	2 (15.4)	3 (15)	544 (9.9)	4061 (15.1)	<0.0001
Conjunctivitis	1493 (9.3)	0 (0)	2 (5.4)	4 (4.3)	487 (9.3)	0 (0)	1 (5)	304 (5.6)	2291 (8.5)	<0.0001
Cyanosis	1618 (10.1)	0 (0)	3 (8.1)	3 (3.3)	664 (12.7)	2 (15.4)	1 (5)	367 (6.7)	2658 (9.9)	<0.0001
Sudden onset of symptoms	5259 (32.7)	3 (50)	3 (8.1)	47 (51.1)	2472 (47.3)	7 (53.8)	9 (45)	2022 (36.9)	9822 (36.4)	<0.0001

Data expressed as frequency (%). P values were calculated by X^2 or One-way ANOVA.

a: Statistical difference with respect to No antiviral/antibiotic
SD: Standard deviation

EXCLI Journal 2021;20:199-222 – ISSN 1611-2156
Received: January 19, 2021, accepted: February 01, 2021, published: February 04, 2021

Supplementary Table 3: Baseline and follow-up characteristics of laboratory confirmed COVID-19 survivors and non-survivors who received medical attention in 688 accredited units in Mexico City between February 24, 2020 and September 14, 2020

	**Total population n=136855**	**Survivors n=125176**	**Non-survivors n=11679**	**p Value**
Sex				
Women	66683 (48.7)	62907 (50.3)	3776 (32.3)	<0.0001
Men	70172 (51.3)	62269 (49.7)	7903 (67.7)	
Age, mean (SD)	44.2 (16.8)	42.5 (15.9)	62 (14.1)	<0.0001
Age categories				
0-19 years	7558 (5.5)	7514 (6)	44 (0.4)	<0.0001
20-29 years	20098 (14.7)	19974 (16)	124 (1.1)	
30-39 years	29434 (21.5)	28909 (23.1)	525 (4.5)	
40-49 years	29553 (21.6)	28084 (22.4)	1469 (12.6)	
50-59 years	24928 (18.2)	22298 (17.8)	2630 (22.5)	
60-69 years	15070 (11)	11840 (9.5)	3230 (27.7)	
70-79 years	7183 (5.2)	4731 (3.8)	2452 (21)	
80-89 years	2594 (1.9)	1540 (1.2)	1054 (9)	
90-99 years	419 (0.3)	270 (0.2)	149 (1.3)	
≥100 years	18 (0.01)	16 (0.01)	2 (0.001)	
Indigenous self-identification	713 (0.5)	639 (0.5)	74 (0.6)	0.08
Occupation				
Technical services	1916 (1.4)	1702 (1.4)	214 (1.8)	<0.0001
Education	10006 (7.3)	9923 (7.9)	83 (0.7)	
Healthcare	17281 (12.6)	16954 (13.5)	327 (2.8)	
Agricultural activities	302 (0.2)	244 (0.2)	58 (0.5)	
Commerce	50450 (36.9)	47070 (37.6)	3380 (28.9)	
Other	24630 (18)	22383 (17.9)	2247 (19.2)	
Unemployed	5685 (4.2)	4539 (3.6)	1146 (9.8)	
Stay-at-home	26585 (19.4)	22361 (17.9)	4224 (36.2)	
Last-season flu vaccination	25691 (19.7)	24570 (20.7)	1121 (9.6)	<0.0001
Special populations				
Pregnancy	583 (0.9)	573 (0.9)	10 (0.3)	<0.0001
Age during pregnancy, mean (SD)	29.8 (7.4)	29.8 (7.5)	32.7 (6)	0.2
Last-season flu vaccination	161 (27.6)	158 (27.6)	3 (30)	0.9
Pregnancy Age				
Early adolescent (≤14 years)	2 (0.3)	2 (0.3)	0 (0)	0.2
Late adolescent (15-19 years)	34 (5.8)	34 (5.9)	0 (0)	

EXCLI Journal 2021;20:199-222 – ISSN 1611-2156
Received: January 19, 2021, accepted: February 01, 2021, published: February 04, 2021

	Total population n=136855	Survivors n=125176	Non-survivors n=11679	p Value
Normal age (20-34 years)	404 (69.3)	398 (69.5)	6 (60)	
Advanced maternal age (≥35 years)	143 (24.5)	139 (24.3)	4 (40)	
Trimester of pregnancy				
First trimester	114 (19.6)	114 (19.9)	0 (0.0)	<0.0001
Second trimester	177 (30.4)	174 (30.4)	3 (30)	
Third trimester	292 (50.1)	285 (49.7)	7 (70)	
Puerperium	103 (0.2)	102 (0.2)	1 (0.03)	0.9
Days of puerperium				
1 day	33 (32)	32 (31.4)	1 (100)	0.2
2-7 days	33 (32)	33 (32.4)	0 (0)	
8-42 days	37 (35.9)	37 (36.3)	0 (0)	
Age during puerperium, mean (SD)	31.9 (9.7)	31.9 (9.7)	24	0.5
Last-season flu vaccination	22 (21.4)	22 (21.6)	0 (0)	0.6
Children and adolescents (<18 years)	5791 (4.2)	5751 (4.6)	40 (0.3)	<0.0001
Age, mean (SD)	10.9 (5.2)	10.9 (5.2)	7.5 (6.4)	<0.0001
Last-season flu vaccination	1213 (20.9)	1211 (21.1)	2 (5)	0.01
Non-pregnant/puerperal adults (≥18 years)	130378 (95.3)	118750 (94.9)	11628 (99.6)	<0.0001
Age, mean (SD)	45.7 (15.5)	44.1 (14.7)	62.2 (13.7)	<0.0001
Last-season flu vaccination	25691 (19.7)	24570 (20.7)	1121 (9.6)	<0.0001
Comorbidities				
Diabetes	18229 (13.3)	14136 (11.3)	4093 (35)	<0.0001
COPD	1741 (1.3)	1184 (0.9)	557 (4.8)	<0.0001
Asthma	3035 (2.2)	2858 (2.3)	177 (1.5)	<0.0001
Immunosuppression	1758 (1.3)	1380 (1.1)	378 (3.2)	<0.0001
Hypertension	22185 (16.2)	17574 (14)	4611 (39.5)	<0.0001
HIV/AIDS	573 (0.4)	509 (0.4)	64 (0.5)	0.02
Cardiovascular disease	2724 (2)	2128 (1.7)	596 (5.1)	<0.0001
Obesity	23848 (17.4)	21072 (16.8)	2776 (23.8)	<0.0001
Chronic kidney disease	2067 (1.5)	1298 (1)	769 (6.6)	<0.0001
Smoker	14727 (10.8)	13437 (10.7)	1290 (11)	0.3
Time from symptom onset to medical attention	4.5 (3.9)	4.5 (3.8)	4.9 (3.9)	<0.0001
Baseline symptoms				
Fever	83120 (60.7)	73561 (58.8)	9559 (81.8)	<0.0001
Cough	96206 (70.3)	86484 (69.1)	9722 (83.2)	<0.0001
Sore throat	59040 (43.1)	54325 (43.4)	4715 (40.4)	<0.0001

	Total population n=136855	Survivors n=125176	Non-survivors n=11679	p Value
Shortness of breath	42942 (31.4)	33762 (27)	9180 (78.6)	<0.0001
Irritability	24098 (17.6)	21767 (17.4)	2331 (20)	<0.0001
Diarrhea	31649 (23.1)	29141 (23.3)	2508 (21.5)	<0.0001
Chest pain	36851 (26.9)	32498 (26)	4353 (37.3)	<0.0001
Chills	48282 (35.3)	43660 (34.9)	4622 (39.6)	<0.0001
Headache	95018 (69.4)	86872 (69.4)	8146 (69.7)	0.4
Myalgias	70666 (51.6)	63547 (50.8)	7119 (61.0)	<0.0001
Arthralgias	64381 (47)	57539 (46)	6842 (58.6)	<0.0001
Abrupt deterioration	62460 (45.6)	54794 (43.8)	7666 (65.6)	<0.0001
Rhinorrhea	38288 (28)	35677 (28.5)	2611 (22.4)	<0.0001
Polypnea	15868 (11.6)	12283 (9.8)	3585 (30.7)	<0.0001
Vomit	10123 (7.4)	8956 (7.2)	1167 (10)	<0.0001
Abdominal pain	17338 (12.7)	15639 (12.5)	1699 (14.5)	<0.0001
Conjunctivitis	16941 (12.4)	15965 (12.8)	976 (8.4)	<0.0001
Cyanosis	5917 (4.3)	4430 (3.5)	1487 (12.7)	<0.0001
Sudden onset of symptoms	46723 (34.1)	42636 (34.1)	4087 (35)	0.04
Contact with confirmed COVID-19 case	70923 (51.8)	69506 (55.5)	1417 (12.1)	<0.0001
Type of treatment received				
No antiviral / antibiotic	114143 (83.4)	106792 (85.3)	7351 (62.9)	<0.0001
Antiviral only	4044 (3)	2980 (2.4)	1064 (9.1)	
Antibiotic only	13743 (10)	11788 (9.4)	1955 (16.7)	
Antiviral + antibiotic	4925 (3.6)	3616 (2.9)	1309 (11.2)	
Type of antiviral used				
Acyclovir	36 (0.03)	32 (0.03)	4 (0.03)	<0.0001
Amantadine	319 (0.2)	304 (0.2)	15 (0.1)	
Lopinavir-Ritonavir	100 (0.1)	75 (0.1)	25 (0.2)	
Oseltamivir	8414 (6.1)	6100 (4.9)	2314 (19.8)	
Rimantadine	61 (0.04)	54 (0.04)	7 (0.1)	
Zanamivir	39 (0.03)	31 (0.02)	8(0.1)	

Data expressed as frequency (%) or median (SD). P values were calculated by X^2 or Student's t-test.

SD: Standard deviation, COPD: Chronic obstructive pulmonary disease, HIV/AIDS: Human immunodeficiency virus/acquired immune deficiency syndrome

EXCLI Journal 2021;20:199-222 – ISSN 1611-2156
Received: January 19, 2021, accepted: February 01, 2021, published: February 04, 2021

Supplementary Table 4: Survival analysis of laboratory-confirmed COVID-19 patients who underwent invasive mechanical ventilation (IMV) and were treated with antivirals and/or antibiotics in one of 197 accredited hospitals in Mexico City, between February 24, 2020 and September 14, 2020

Group	Total	Deaths	Survivors	Survival	p value
Patients receiving IMV	6752	5346	1406	20.8%	-
Type of treatment					
No antiviral/antibiotic	3931	3240	691	17.6%	Reference
Antiviral only	507	431	76	15.0%	0.001
Antibiotic only	1586	1087	499	31.5%	<0.0001
Antiviral + Antibiotic	768	621	147	19.1%	0.3
Type of antiviral or antibiotic only					
No antiviral/antibiotic	3931	3240	691	17.6%	Reference
Acyclovir	2	2	0	0.0%	0.6
Amantadine	7	7	0	0.0%	0.5
Lopinavir-Ritonavir	23	16	7	30.4%	0.1
Oseltamivir	1235	1019	216	17.5%	0.2
Rimantadine	3	3	0	0.0%	0.4
Zanamivir	5	5	0	0.0%	0.9
Antibiotic only	1586	1087	499	31.5%	<0.0001
Treatment with oseltamivir					
No antiviral/antibiotic	3931	3240	691	17.6%	Reference
Oseltamivir	488	413	75	15.4%	0.01
Antibiotic only	1586	1087	499	31.5%	<0.0001
Oseltamivir + Antibiotic	747	606	141	18.9%	0.4

Data expressed as number of patients or percentual of survival. p values were calculated by Log-Rank test.

EXCLI Journal 2021;20:199-222 – ISSN 1611-2156
Received: January 19, 2021, accepted: February 01, 2021, published: February 04, 2021

Supplementary Table 5: Survival analysis of laboratory-confirmed COVID-19 patients treated with antivirals and/or antibiotics and admitted to one of 125 accredited intensive care units (ICU) in Mexico City, between February 24, 2020 and September 14, 2020

Group	Total	Deaths	Survivors	Survival	p value
Patients admitted to ICU	2425	1475	950	39.2%	-
Type of treatment					
No antiviral/antibiotic	908	494	414	45.6%	Reference
Antiviral only	77	53	24	31.2%	0.001
Antibiotic only	1011	610	401	39.7%	0.02
Antiviral + Antibiotic	461	336	125	27.1%	<0.0001
Antiviral or antibiotic only					
No antiviral/antibiotic	908	494	414	45.6%	Reference
Acyclovir	1	1	0	0.0%	0.08
Amantadine	2	0	2	100.0%	0.20
Lopinavir-Ritonavir	29	17	12	41.4%	0.40
Oseltamivir	506	371	135	26.7%	<0.0001
Antibiotic only	1011	610	401	39.7%	0.02
Oseltamivir					
No antiviral/antibiotic	908	494	414	45.6%	Reference
Oseltamivir	66	48	18	27.3%	<0.0001
Antibiotic only	1011	610	401	39.7%	0.02
Oseltamivir + Antibiotic	440	323	117	26.6%	<0.0001

Data expressed as number of patients or percentual of survival. p values were calculated by Log-Rank test.

Received: January 19, 2021, accepted: February 01, 2021, published: February 04, 2021

Supplementary Table 6: Survival analysis of laboratory-confirmed COVID-19 non-pregnant/puerperal adults treated with antivirals and/or antibiotics in 379 accredited medical units in Mexico City, between February 24, 2020 and September 14, 2020

Group	Total	Deaths	Survivors	Survival	p value
Non-pregnant/puerperal adults	129837	11569	118268	91.1%	-
Type of treatment					
No antiviral/antibiotic	108213	7314	100899	93.2%	Reference
Antiviral only	3990	1063	2927	73.4%	<0.0001
Antibiotic only	13300	1945	11355	85.4%	<0.0001
Antiviral + Antibiotic	4875	1306	3569	73.2%	<0.0001
Type of antiviral or antibiotic only					
No antiviral/antibiotic	108213	7314	100899	93.2%	Reference
Acyclovir	36	4	32	88.9%	0.4
Amantadine	308	15	293	95.1%	0.2
Lopinavir-Ritonavir	99	25	74	74.7%	<0.0001
Oseltamivir	8324	2310	6014	72.2%	<0.0001
Rimantadine	59	7	52	88.1%	0.1
Zanamivir	39	8	31	79.5%	0.001
Antibiotic only	13300	1945	11355	85.4%	<0.0001
Treatment with oseltamivir					
No antiviral / Antibiotic	108213	7314	100899	93.2%	Reference
Oseltamivir	3738	1032	2706	72.4%	<0.0001
Antibiotic only	13300	1945	11355	85.4%	<0.0001
Oseltamivir + Antibiotic	4586	1278	3308	72.1%	<0.0001

Data expressed as number of patients or percentual of survival. p values were calculated by Log-Rank test.

EXCLI Journal 2021;20:199-222 – ISSN 1611-2156
Received: January 19, 2021, accepted: February 01, 2021, published: February 04, 2021

Supplementary Table 7: Survival analysis of laboratory-confirmed COVID-19 children and adolescents treated with antivirals and/or antibiotics in 273 accredited medical units in Mexico City, between February 24, 2020 and September 14, 2020

Group	Total	Deaths	Survivors	Survival	p value
Children and adolescents	5791	40	5751	99.31%	-
Type of treatment					
No antiviral/antibiotic	5336	28	5308	99.5%	Reference
Antiviral only	40	0	40	100.0%	0.7
Antibiotic only	376	10	366	97.3%	<0.0001
Antiviral + Antibiotic	39	2	37	94.9%	<0.0001
Type of antiviral or antibiotic only					
No antiviral/antibiotic	5336	28	5308	99.5%	Reference
Amantadine	9	0	9	100.0%	0.8
Lopinavir-Ritonavir	1	0	1	100.0%	0.9
Oseltamivir	67	2	65	97.0%	0.008
Rimantadine	2	0	2	100.0%	0.9
Antibiotic only	376	10	366	97.3%	<0.0001
Treatment with oseltamivir					
No antiviral/antibiotic	5336	28	5308	99.5%	Reference
Oseltamivir	36	0	36	100.0%	0.7
Antibiotic only	376	10	366	97.3%	<0.0001
Oseltamivir + Antibiotic	31	2	29	93.5%	<0.0001

Data expressed as number of patients or percentual of survival. p values were calculated by Log-Rank test.

EXCLI Journal 2021;20:199-222 – ISSN 1611-2156
Received: January 19, 2021, accepted: February 01, 2021, published: February 04, 2021

Supplementary Table 8: Survival analysis of laboratory-confirmed COVID-19 pregnant women treated with antivirals and/or antibiotics in 177 accredited medical units in Mexico City, between February 24, 2020 and September 14, 2020

Group	Total	Deaths	Survivors	Survival	p value
Pregnancy	581	10	571	98.3%	-
Type of treatment					
No antiviral / Antibiotic	530	8	522	98.5%	Reference
Antiviral only	12	1	11	91.7%	0.07
Antibiotic only	35	0	35	100.0%	0.9
Antiviral + Antibiotic	6	1	5	83.3%	0.004
Type of antiviral					
No antiviral / Antibiotic	530	8	522	98.5%	Reference
Amantadine	2	0	2	100.0%	0.9
Oseltamivir	16	2	14	87.5%	0.001
Antibiotic only	35	0	35	100.0%	0.5
Treatment with Oseltamivir					
No antiviral / Antibiotic	530	8	522	98.5%	Reference
Oseltamivir	11	1	10	90.9%	0.05
Antibiotic only	35	0	35	100.0%	0.5
Oseltamivir + Antibiotic	5	1	4	80.0%	0.001

Data expressed as number of patients or percentual of survival. p values were calculated by Log-Rank test.

EXCLI Journal 2021;20:199-222 – ISSN 1611-2156
Received: January 19, 2021, accepted: February 01, 2021, published: February 04, 2021

Supplementary Table 9: Unadjusted mortality risk in laboratory-confirmed COVID-19 patients receiving antivirals, antibiotics, both, or none in accredited COVID-19 medical units in Mexico City

	All patients		Ambulatory		Hospitalized		Non-Critical		Critical	
	HR (95% CI)	p	HR (95% CI)	p	HR (95% CI)	p	HR (95% CI)	p	HR (95% CI)	p
**Models for type of treatment**										
No antiviral / antibiotic	Reference		Reference		Reference		Reference		Reference	
Antiviral only	4.43 (4.15-4.72)	<0.0001	7.48 (6.29-8.90)	<0.0001	1.19 (1.11-1.27)	<0.0001	5.07 (4.67-5.52)	<0.0001	1.21 (1.09-1.34)	<0.0001
Antibiotic only	2.15 (2.05-2.27)	<0.0001	1.09 (0.88-1.36)	0.4	0.79 (0.75-0.83)	<0.0001	1.89 (1.75-2.03)	<0.0001	0.63 (0.58-0.67)	<0.0001
Antiviral + antibiotic	4.31 (4.05-4.58)	<0.0001	4.02 (3.09-5.22)	<0.0001	0.90 (0.85-0.96)	0.001	4.65 (4.29-5.05)	<0.0001	0.96 (0.88-1.05)	0.4
**Models for type of antiviral**										
No antiviral / antibiotic	Reference		Reference		Reference		Reference		Reference	
Acyclovir	1.71 (0.64-4.56)	0.3	Not estimable		1.68 (0.63-4.48)	0.3	1.51 (0.38-6.07)	0.6	1.51 (0.38-6.03)	0.6
Amantadine	0.71 (0.43-1.17)	0.2	1.06 (0.34-3.29)	0.9	0.77 (0.44-1.36)	0.4	0.68 (0.34-1.36)	0.3	0.85 (0.40-1.78)	0.7
Lopinavir-Ritonavir	3.90 (2.61-5.83)	<0.0001	12.96 (1.83-92.15)	0.01	0.57 (0.38-0.85)	.006	2.69 (1.28-5.65)	0.009	0.57 (0.36-0.90)	0.02
Oseltamivir	4.57 (4.36-4.79)	<0.0001	6.55 (5.63-7.63)	<0.0001	1.01 (0.97-1.07)	0.6	5.12 (4.81-5.46)	<0.0001	1.07 (0.99-1.14)	0.08
Rimantadine	1.74 (0.84-3.68)	0.1	2.02 (0.28-14.36)	0.5	1.19 (0.54-2.65)	0.7	1.83 (0.69-4.89)	0.2	1.77 (0.57-5.48)	0.3
Zanamivir	3.22 (1.61-6.44)	0.001	5.24 (0.73-37.23)	0.09	0.76 (0.36-1.60)	0.5	2.34 (0.76-7.27)	0.1	1.18 (0.49-2.85)	0.7
Antibiotic only	2.15 (2.05-2.27)	<0.0001	1.09 (0.88-1.36)	0.4	0.79 (0.75-0.83)	<0.0001	1.89 (1.75-2.03)	<0.0001	0.63 (0.59-0.67)	<0.0001
**Models for Acyclovir**										
No antiviral / antibiotic	Reference		Reference		Reference		Reference		Reference	
Acyclovir only	2.16 (0.30-15.31)	0.4	Not estimable		4.09 (0.58-29.05)	0.2	Not estimable		2.21 (0.31-15.68)	0.4
Antibiotic only	2.15 (2.05-2.27)	<0.0001	1.09 (0.88-1.36)	0.4	0.78 (0.75-0.83)	<0.0001	1.89 (1.75-2.03)	<0.0001	0.63 (0.58-0.67)	<0.0001
Acyclovir + antibiotic	1.60 (0.52-4.97)	0.4	Not estimable		1.41 (0.45-4.37)	0.6	1.85 (0.46-7.38)	0.4	1.16 (0.16-8.24)	0.9
**Models for Amantadine**										
No antiviral / antibiotic	Reference		Reference		Reference		Reference		Reference	
Amantadine only	1.17 (0.68-2.02)	0.6	1.34 (0.34-5.38)	0.7	1.47 (0.82-2.66)	0.2	1.15 (0.55-2.42)	0.7	1.12 (0.50-2.50)	0.8
Antibiotic only	2.23 (2.12-2.34)	<0.0001	1.09 (0.88-1.36)	0.4	0.79 (0.75-0.83)	<0.0001	1.89 (1.75-2.03)	<0.0001	0.63 (0.58-0.67)	<0.0001
Amantadine + antibiotic	0.19 (0.05-0.80)	0.02	0.74 (0.10-5.27)	0.8	0.13 (0.09-0.88)	0.04	0.18 (0.03-1.25)	0.08	0.34 (0.05-2.44)	0.3

EXCLI Journal 2021;20:199-222 – ISSN 1611-2156
Received: January 19, 2021, accepted: February 01, 2021, published: February 04, 2021

	All patients		Ambulatory		Hospitalized		Non-Critical		Critical	
	HR (95% CI)	p	HR (95% CI)	p	HR (95% CI)	p	HR (95% CI)	p	HR (95% CI)	p
**Models for Lopinavir-Ritonavir**										
No antiviral / antibiotic	Reference		Reference		Reference		Reference		Reference	
Lopinavir-Ritonavir only	2.79 (1.05-7.44)	0.04	424.97 (59.62-3029)	<0.0001	0.38 (0.14-1.00)	0.05	1.91 (0.27-13.63)	0.5	0.36 (0.14-0.96)	0.04
Antibiotic only	2.15 (2.05-2.27)	<0.0001	1.09 (0.88-1.36)	0.4	0.79 (0.75-0.83)	<0.0001	1.89 (1.75-2.03)	<0.0001	0.63 (0.58-0.67)	<0.0001
Lopinavir-Ritonavir + antibiotic	4.24 (2.73-6.58)	<0.0001	Not estimable		0.64 (0.41-0.99)	0.04	2.88 (1.29-6.42)	0.01	0.67 (0.39-1.14)	0.1
**Models for Oseltamivir**										
No antiviral / antibiotic	Reference		Reference		Reference		Reference		Reference	
Oseltamivir only	4.62 (4.33-4.94)	<0.0001	8.00 (6.71-9.54)	<0.0001	1.20 (1.12-1.29)	<0.0001	5.36 (4.92-5.83)	<0.0001	1.24 (1.12-1.37)	<0.0001
Antibiotic only	2.15 (2.05-2.27)	<0.0001	1.09 (0.88-1.36)	0.4	0.79 (0.75-0.83)	<0.0001	1.89 (1.75-2.03)	<0.0001	0.63 (0.59-0.67)	<0.0001
Oseltamivir + antibiotic	4.53 (4.26-4.82)	<0.0001	4.56 (3.51-5.94)	<0.0001	0.91 (0.86-0.97)	0.003	4.92 (4.54-5.35)	<0.0001	0.97 (0.89-1.06)	0.5
**Models for Rimantadine**										
No antiviral / antibiotic	Reference		Reference		Reference		Reference		Reference	
Rimantadine only	2.75 (1.24-6.12)	0.01	4.09 (0.58-29.09)	0.2	1.21 (0.50-2.90)	0.7	2.54 (0.82-7.88)	0.1	1.79 (0.58-5.55)	0.3
Antibiotic only	2.15 (2.05-2.27)	<0.0001	1.09 (0.88-1.36)	0.4	0.78 (0.75-0.83)	<0.0001	1.88 (1.75-2.03)	<0.0001	0.63 (0.58-0.67)	<0.0001
Rimantadine + antibiotic	0.55 (0.08-3.91)	0.6	Not estimable		1.14 (0.16-8.06)	0.9	0.99 (0.14-7.08)	0.9	-	-
**Models for Zanamivir**										
No antiviral / antibiotic	Reference		Reference		Reference		Reference		Reference	
Zanamivir only	3.91 (1.75-8.69)	0.001	10.31 (1.45-73.28)	0.02	0.78 (0.33-1.88)	0.6	1.40 (0.19-9.96)	0.7	1.19 (0.49-2.86)	0.7
Antibiotic only	2.15 (2.05-2.28)	<0.0001	1.09 (0.88-1.36)	0.4	0.79 (0.75-0.83)	<0.0001	1.89 (1.75-2.03)	<0.0001	0.63 (0.49-2.86)	<0.0001
Zanamivir + antibiotic	2.11 (0.53-8.43)	0.3	Not estimable		0.72 (0.18-2.87)	0.6	3.52 (0.88-14.10)	0.07	-	-

HR: Hazard ratio, 95%CI: 95% confidence intervals

EXCLI Journal 2021;20:199-222 – ISSN 1611-2156
Received: January 19, 2021, accepted: February 01, 2021, published: February 04, 2021

Supplementary Table 10: Mortality risk in laboratory-confirmed COVID-19 patients who underwent invasive mechanical ventilation (IMV) and were treated with antivirals and/or antibiotics in one of 197 accredited hospitals in Mexico City, between February 24, 2020 and September 14, 2020

	Unadjusted model		Adjusted model[a]	
	HR (95% CI)	p value	HR (95% CI)	p value
**Type of treatment**				
No antiviral/antibiotic	Reference		Reference	
Antiviral only	1.18 (1.07-1.31)	0.001	1.13 (1.02-1.25)	0.02
Antibiotic only	0.62 (0.59-0.67)	<0.0001	0.64 (0.60-0.69)	<0.0001
Antiviral + antibiotic	0.95 (0.88-1.04)	0.3	0.98 (0.90-1.07)	0.7
**Type of antiviral**				
No antiviral/antibiotic	Reference		Reference	
Acyclovir	1.42 (0.36-5.68)	0.6	1.96 (0.49-7.83)	0.3
Amantadine	1.29 (0.61-2.70)	0.5	1.29 (0.61-2.72	0.5
Lopinavir-Ritonavir	0.67 (0.41-1.09)	0.1	0.68 (0.42-1.12)	0.1
Oseltamivir	1.04 (0.97-1.12)	0.3	1.04 (0.97-1.12)	0.2
Rimantadine	1.66 (0.53-5.14)	0.4	1.57 (0.50-4.90)	0.4
Zanamivir	1.09 (0.48-2.64)	0.8	0.87 (0.36-2.09)	0.8
Antibiotic only	0.63 (0.59-0.67)	<0.0001	0.64 (0.60-0.69)	<0.0001
**Acyclovir**				
No antiviral/antibiotic	Reference		Reference	
Acyclovir only	2.09 (0.29-14.9)	0.5	2.85 (0.40-20.35)	0.3
Antibiotic only	0.63 (0.58-0.67)	<0.0001	0.64 (0.60-0.69)	<0.0001
Acyclovir + antibiotic	1.09 (0.15-7.74)	0.9	1.52 (0.21-10.78)	0.7
**Amantadine**				
No antiviral/antibiotic	Reference		Reference	
Amantadine only	1.51 (0.68-3.35)	0.3	1.50 (0.67-3.35)	0.32
Antibiotic only	0.63 (0.58-0.67)	<0.0001	0.64 (0.6-0.69)	<0.0001
Amantadine + antibiotic	0.71 (0.09-5.01)	0.7	0.71 (0.10-5.09)	0.7
**Lopinavir-Ritonavir**				
No antiviral/antibiotic	Reference		Reference	
Lopinavir-Ritonavir only	0.59 (0.19-1.86)	0.4	0.53 (0.17-1.63)	0.3
Antibiotic only	0.63 (0.58-0.67)	<0.0001	0.64 (0.60-0.69)	<0.0001
Lopinavir-Ritonavir + antibiotic	0.68 (0.39-1.17)	0.2	0.74 (0.43-1.27)	0.3
**Oseltamivir**				
No antiviral/antibiotic	Reference		Reference	
Oseltamivir only	1.19 (1.07-1.31)	0.001	1.14 (1.02-1.26)	0.02
Antibiotic only	0.63 (0.59-0.67)	<0.0001	0.64 (0.60-0.69)	<0.0001
Oseltamivir + antibiotic	0.96 (0.88-1.05)	0.4	0.99 (0.91-1.08)	0.8
**Rimantadine**				
No antiviral/antibiotic	Reference		Reference	
Rimantadine only	1.68 (0.54-5.20)	0.4	1.66 (0.53-5.17)	0.4
Antibiotic only	0.63 (0.58-0.67)	<0.0001	0.64 (0.60-0.69)	<0.0001
**Zanamivir**				
No antiviral/antibiotic	Reference		Reference	
Zanamivir only	1.10 (0.46-2.66)	0.8	0.87 (0.36-2.10)	.80
Antibiotic only	0.63 (0.58-0.67)	<0.0001	0.64 (0.60-0.69)	<0.0001

HR: Hazard ratio, 95% CI: 95% confident interval.

a: Models adjusted by: Sex (men), Age, Diabetes, Chronic obstructive pulmonary disease, Immunosuppression, Hypertension, Cardiovascular disease, Chronic kidney disease, Smoker, Unemployed, Irritability, Headache, Myalgias, Arthralgias, Rhinorrhea, Abdominal pain, Cyanosis

EXCLI Journal 2021;20:199-222 – ISSN 1611-2156
Received: January 19, 2021, accepted: February 01, 2021, published: February 04, 2021

Supplementary Table 11: Mortality risk in laboratory-confirmed COVID-19 patients who were treated with antivirals and/or antibiotics and admitted to one of 125 accredited intensive care units (ICU) in Mexico City, between February 24, 2020 and September 14, 2020

	Unadjusted model		Adjusted model[a]	
	HR (95% CI)	p value	HR (95% CI)	p value
**Type of treatment**				
No antiviral/antibiotic	Reference		Reference	
Antiviral only	1.60 (1.21-2.13)	0.001	1.42 (1.07-1.89)	0.02
Antibiotic only	1.15 (1.02-1.29)	0.02	1.05 (0.93-1.19)	0.4
Antiviral + antibiotic	1.71 (1.49-1.97)	<0.0001	1.49 (1.30-1.72)	<0.0001
**Type of antiviral**				
No antiviral/antibiotic	Reference		Reference	
Acyclovir	5.33 (0.75-37.9)	0.09	8.38 (1.16-60.62)	0.04
Amantadine	Not estimable		Not estimable	
Lopinavir-Ritonavir	1.21 (0.75-1.97)	0.44	0.99 (0.61-1.61)	0.9
Oseltamivir	1.74 (1.52-1.96)	<0.0001	1.52 (1.33-1.74)	<0.0001
Antibiotic only	1.15 (1.02-1.29)	0.02	1.05 (0.93-1.18)	0.4
**Acyclovir**				
No antiviral/antibiotic	Reference		Reference	
Acyclovir only	5.46 (0.77-38.9)	0.09	10.21 (1.40-74.38)	0.02
Antibiotic only	1.15 (1.02-1.29)	0.02	1.05 (0.93-1.18)	0.4
**Amantadine**				
No antiviral/antibiotic	Reference		Reference	
Amantadine only	Not estimable		Not estimable	
Antibiotic only	1.15 (1.02-1.29)	0.02	1.05 (0.93-1.18)	0.4
Amantadine + antibiotic	Not estimable		Not estimable	
**Lopinavir-Ritonavir**				
No antiviral/antibiotic	Reference		Reference	
Lopinavir-Ritonavir only	0.74 (0.28-1.98)	0.6	0.50 (0.19-1.35)	0.2
Antibiotic only	1.15 (1.02-1.29)	0.02	1.05 (0.93-1.18)	0.5
Lopinavir-Ritonavir + antibiotic	1.50 (0.89-2.61)	0.2	1.32 (0.76-2.29)	0.3
**Oseltamivir**				
No antiviral/antibiotic	Reference		Reference	
Oseltamivir only	1.79 (1.34-2.42)	<0.0001	1.64 (1.22-2.21)	<0.0001
Antibiotic only	1.15 (1.02-1.29)	0.02	1.05 (0.93-1.19)	0.4
Oseltamivir + antibiotic	1.73 (1.51-1.99)	<0.0001	1.50 (1.30-1.73)	<0.0001

HR: Hazard ratio, 95% CI: 95% confident interval.

a: Models adjusted by: Sex (men), Age, Diabetes, Chronic obstructive pulmonary disease, Hypertension, Obesity, Chronic kidney disease, Unemployed, Fever, Cough, Shortness of breath, Myalgias, Vomit, Cyanosis

EXCLI Journal 2021;20:199-222 – ISSN 1611-2156
Received: January 19, 2021, accepted: February 01, 2021, published: February 04, 2021

Supplementary Table 12: Mortality risk in laboratory-confirmed COVID-19 non-pregnant/puerperal adults treated with antivirals and/or antibiotics in 379 accredited medical units in Mexico City, between February 24, 2020 and September 14, 2020

	Unadjusted model		Adjusted model[a]	
	HR (95% CI)	p value	HR (95% CI)	p value
**Type of treatment**				
No antiviral/antibiotic	Reference		Reference	
Antiviral only	4.34 (4.07-4.62)	<0.0001	1.80 (1.68-1.92)	<0.0001
Antibiotic only	2.18 (2.08-2.29)	<0.0001	1.19 (1.13-1.25)	<0.0001
Antiviral + antibiotic	4.31 (4.07-4.57)	<0.0001	1.64 (1.54-1.74)	<0.0001
**Type of antiviral**				
No antiviral/antibiotic	Reference		Reference	
Acyclovir	1.57 (0.59-4.18)	0.4	1.40 (0.52-3.72)	0.5
Amantadine	0.69 (0.42-1.16)	0.2	0.72 (0.43-1.20)	0.2
Lopinavir-Ritonavir	3.88 (2.62-5.74)	<0.0001	1.14 (0.77-1.69)	0.5
Oseltamivir	4.52 (4.32-4.74)	<0.0001	1.73 (1.65-1.82)	<0.0001
Rimantadine	1.75 (0.83-3.67)	0.1	1.47 (0.70-3.08)	0.3
Zanamivir	3.04 (1.52-6.08)	0.002	1.61 (0.81-3.22)	0.2
Antibiotic only	2.18 (2.08-2.29)	<0.0001	1.19 (1.13-1.25)	<0.0001
**Acyclovir**				
No antiviral/antibiotic	Reference		Reference	
Acyclovir only	2.09 (0.29-14.8)	0.5	7.50 (1.06-53.27)	0.04
Antibiotic only	2.18 (2.08-2.29)	<0.0001	1.16 (1.10-1.22)	<0.0001
Acyclovir + antibiotic	1.45 (0.46-4.48)	0.5	1.11 (0.36-3.45)	0.9
**Amantadine**				
No antiviral/antibiotic	Reference		Reference	
Amantadine only	1.15 (0.67-1.98)	0.6	1.75 (1.02-3.02)	0.04
Antibiotic only	2.18 (2.08-2.29)	<0.0001	1.16 (1.10-1.22)	<0.0001
Amantadine + antibiotic	0.19 (0.05-0.78)	0.02	0.15 (0.04-0.59)	0.007
**Lopinavir-Ritonavir**				
No antiviral/antibiotic	Reference		Reference	
Lopinavir-Ritonavir only	3.27 (1.36-7.88)	0.008	0.83 (0.34-1.99)	0.7
Antibiotic only	2.18 (2.08-2.29)	<0.0001	1.16 (1.11-1.22)	<0.0001
Lopinavir-Ritonavir + antibiotic	4.06 (2.62-6.29)	<0.0001	1.18 (0.76-1.83)	0.5
**Oseltamivir**				
No antiviral/antibiotic	Reference		Reference	
Oseltamivir only	4.52 (4.24-4.83)	<0.0001	1.81 (1.69-1.93)	<0.0001
Antibiotic only	2.18 (2.08-2.29)	<0.0001	1.19 (1.13-1.25)	<0.0001
Oseltamivir + antibiotic	4.52 (4.26-4.79)	<0.0001	1.68 (1.58-1.78)	<0.0001
**Rimantadine**				
No antiviral/antibiotic	Reference		Reference	
Rimantadine only	2.71 (1.22-6.03)	0.02	2.02 (0.91-4.50)	0.1
Antibiotic only	2.18 (2.08-2.29)	<0.0001	1.16 (1.11-1.22)	<0.0001
Rimantadine + antibiotic	0.56 (0.08-3.97)	0.6	0.53 (0.07-3.77)	0.5
**Zanamivir**				
No antiviral/antibiotic	Reference		Reference	
Zanamivir only	3.79 (1.71-8.45)	0.001	2.02 (0.91-4.50)	0.1
Antibiotic only	2.18 (2.08-2.29)	<0.0001	1.16 (1.11-1.22)	<0.0001
Zanamivir + antibiotic	1.90 (0.48-7.62)	0.36	0.97 (0.24-3.86)	0.9

HR: Hazard ratio, 95% CI: 95% confident interval.
a: Models adjusted by: Sex (men), Age, Diabetes, Chronic obstructive pulmonary disease, Immunosuppression, Hypertension, Human immunodeficiency virus/acquired immune deficiency syndrome, Obesity, Cardiovascular disease, Chronic kidney disease, Smoker, Unemployed, Fever, Cough, Shortness of breath, Irritability, Chest pain, Myalgias, Arthralgias, Polypnea, Vomit, Abdominal pain

EXCLI Journal 2021;20:199-222 – ISSN 1611-2156
Received: January 19, 2021, accepted: February 01, 2021, published: February 04, 2021

Supplementary Table 13: Mortality risk in laboratory-confirmed COVID-19 children and adolescents treated with antivirals and/or antibiotics in 273 accredited medical units in Mexico City, between February 24, 2020 and September 14, 2020

	**Unadjusted model**		**Adjusted model[a]**	
	**HR (95% CI)**	**p value**	**HR (95% CI)**	**p value**
**Type of treatment**				
No antiviral/antibiotic	Reference		Reference	
Antiviral only	Not estimable		Not estimable	
Antibiotic only	5.01 (2.43-10.32)	<0.0001	4.22 (2.01-8.86)	<0.0001
Antiviral + antibiotic	9.68 (2.31-40.62)	0.002	6.54 (1.44-29.63)	0.01
**Type of antiviral**				
No antiviral/antibiotic	Reference		Reference	
Amantadine	Not estimable		Not estimable	
Lopinavir-Ritonavir	Not estimable		Not estimable	
Oseltamivir	5.66 (1.35-23.73)	0.02	4.22 (0.94-18.99)	0.06
Rimantadine	Not estimable		Not estimable	
Antibiotic only	5.01 (2.43-10.31)	<0.0001	4.20 (2.00-8.83)	<0.0001
**Oseltamivir**				
No antiviral/antibiotic	Reference		Reference	
Oseltamivir only	Not estimable		Not estimable	
Antibiotic only	5.01 (2.43-10.3)	<0.0001	4.20 (2.00-8.82)	<0.0001
Oseltamivir + antibiotic	12.10 (2.89-50.84)	0.001	7.95 (1.70-37.21)	0.01

HR: Hazard ratio, 95% CI: 95% confident interval.

a: Models adjusted by: Diabetes, Immunosuppression, Chronic kidney disease

Received: January 19, 2021, accepted: February 01, 2021, published: February 04, 2021

Supplementary Table 14: Mortality risk in laboratory-confirmed COVID-19 pregnant women treated with antivirals and/or antibiotics in 117 accredited medical units in Mexico City, between February 24, 2020 and September 14, 2020

	**Non-adjusted model**	
	**HR (95% CI)**	**P value**
**Type of treatment**		
No antiviral/antibiotic	Reference	
Antiviral only	5.51 (0.69-44.06)	0.1
Antibiotic only	Not estimable	
Antiviral + antibiotic	11.14 (1.39-89.07)	0.02
**Type of antiviral**		
No antiviral/antibiotic	Reference	
Amantadine	Not estimable	
Oseltamivir	8.35 (1.77-39.30)	0.007
Antibiotic only	Not estimable	
**Oseltamivir**		
No antiviral/antibiotic	Reference	
Oseltamivir only	6.04 (0.76-48.26)	0.09
Antibiotic only	Not estimable	
Oseltamivir + antibiotic	13.52 (1.69-108.10)	0.01

HR: Hazard ratio, 95% CI: 95% confident interval

EXCLI Journal 2021;20:199-222 – ISSN 1611-2156
Received: January 19, 2021, accepted: February 01, 2021, published: February 04, 2021

Supplementary Table 15: E-Values for Cox regression models in the general population, ambulatory, hospitalized, non-critical, and critical COVID-19 patients

	All patients	Ambulatory	Hospitalized	Non-Critical	Critical
**Models for type of treatment**					
Antiviral only	2.83 (2.60)	8.87 (7.34)	-	3.48 (3.12)	-
antibiotic only	1.51 (1.37)	2.12 (1.5)	1.77 (1.60)	-	2.35 (2.12)
Antiviral + antibiotic	2.52 (2.30)	3.23 (2.3)	1.43 (1.21)	2.64 (2.34)	-
**Models for type of antiviral**					
Acyclovir	-	-	4.94 (1.21)	-	-
Amantadine	-	-	-	-	-
Lopinavir-Ritonavir	-	-	2.78 (1.5)	-	-
Oseltamivir	2.71 (2.54)	6.50 (5.47)	-	3.08 (2.83)	-
Rimantadine	-	-	-	-	-
Zanamivir	-	-	-	-	-
Antibiotic only	1.54 (1.37)	2.12 (1.46)	1.77 (1.60)	-	2.30 (2.12)
**Models for Acyclovir**					
Acyclovir only	15.68 (1.54)	-	17.45 (1.83)	-	-
Antibiotic only	1.46 (1.28)	2.17 (1.5)	1.74 (1.60)	-	2.35 (2.12)
Acyclovir + antibiotic	-	-	-	-	-
**Models for Amantadine**					
Amantadine only	2.96 (1.21)	-	-	-	-
Antibiotic only	1.46 (1.28)	2.17 (1.50)	1.74 (1.60)	-	2.35 (2.12)
Amantadine + antibiotic	12.81 (2.78)	-	-	14.87 (1.46)	-
**Models for Lopinavir-Ritonavir**					
Lopinavir-Ritonavir only	-	113 (15.22)	-	-	-
Antibiotic only	1.46 (1.28)	2.17 (1.50)	1.74 (1.60)	-	2.35 (2.12)
Lopinavir-Ritonavir + antibiotic	-	-	-	-	-
**Models for Oseltamivir**					
Oseltamivir only	2.83 (2.60)	9.05 (7.48)	-	3.52 (3.17)	-
Antibiotic only	1.51 (1.37)	2.12 (1.50)	1.74 (1.60)	-	2.35 (2.12)
Oseltamivir + antibiotic	2.60 (2.39)	3.62 (2.69)	1.39 (1.16)	2.75 (2.47)	-
**Models for Rimantadine**					
Rimantadine only	-	-	-	-	-
Antibiotic only	1.46 (1.28)	2.17 (1.50)	1.74 (1.60)	-	2.35 (2.12)
Rimantadine + antibiotic	-	-	-	-	-
**Models for Zanamivir**					
Zanamivir only	-	-	-	-	-
Antibiotic only	1.46 (1.28)	2.17 (1.50)	1.74 (1.60)	-	2.35 (2.12)
Zanamivir + antibiotic	-	-	-	-	-

Data presented as E-value for point estimate (E-value for lower-limit confidence interval)

EXCLI Journal 2021;20:199-222 – ISSN 1611-2156
Received: January 19, 2021, accepted: February 01, 2021, published: February 04, 2021

Supplementary Table 16: E-Values for Cox regression models in non-pregnant/puerperal adults, children and adolescents, VMI, and ICU patients

	IMV	ICU	Non-pregnant/ puerperal adults	Children and adolescents
**Models for type of treatment**				
Antiviral only	1.51 (1.16)	2.19 (1.34)	3.00 (2.75)	-
Antibiotic only	2.50 (2.26)	-	1.67 (1.51)	7.91 (3.43)
Antiviral + antibiotic	-	2.34 (1.92)	2.66 (2.45)	12.56 (2.24)
**Models for type of antiviral**				
Acyclovir	-	16.24 (1.59)	-	-
Amantadine	-	-	-	-
Lopinavir-Ritonavir	-	-	-	-
Oseltamivir	-	2.41 (1.99)	2.85 (2.69)	-
Rimantadine	-	-	-	-
Zanamivir	-	-	-	-
Antibiotic only	2.50 (2.26)	-	1.67 (1.51)	7.87 (3.41)
**Models for Acyclovir**				
Acyclovir only	-	19.91 (2.15)	14.48 (1.31)	-
Antibiotic only	2.50 (2.26)	-	1.59 (1.43)	-
Acyclovir + antibiotic	-	-	-	-
**Models for Amantadine**				
Amantadine only	-	-	2.9 (1.16)	-
Antibiotic only	2.50 (2.26)	-	1.59 (1.43)	-
Amantadine + antibiotic	-	-	-	-
**Models for Lopinavir-Ritonavir**				
Lopinavir-Ritonavir only	-	-	-	-
Antibiotic only	2.50 (2.26)	-	1.59 (1.43)	-
Lopinavir-Ritonavir + antibiotic	-	-	-	-
**Models for Oseltamivir**				
Oseltamivir only	1.54 (1.16)	2.66 (1.74)	3.02 (2.77)	-
Antibiotic only	2.50 (2.26)	-	1.67 (1.51)	7.87 (3.41)
Oseltamivir + antibiotic	-	2.37 (1.92)	2.75 (2.54)	15.38 (2.79)

Data presented as E-value for point estimate (E-value for lower-limit confidence interval)

EXCLI Journal 2021;20:199-222 – ISSN 1611-2156
Received: January 19, 2021, accepted: February 01, 2021, published: February 04, 2021

Supplementary Table 17: Baseline characteristics of patients with COVID-19 treated with antivirals and propensity score-matched controls not treated with antivirals or antibiotics

	Total n=8088	Antiviral n=4044	Control n=4044
Sex			
Women	3634 (44.9)	1821 45.0	1813 (44.8)
Men	4454 (55.1)	2223 (55.0)	2231 (55.2)
Age, mean (SD)	50.5 (16.5)	50.5 (16.5)	50.5 (16.5)
Age categories			
0-19 years	114 (1.4)	57 (1.4)	57 (1.4)
20-29 years	747 (9.2)	372 (9.2)	375 (9.3)
30-39 years	1411 (17.4)	704 (17.4)	707 (17.5)
40-49 years	1667 (20.6)	830 (20.5)	837 (20.7)
50-59 years	1724 (21.3)	872 (21.6)	852 (21.1)
60-69 years	1264 (15.6)	632 (15.6)	632 (15.6)
70-79 years	821 (10.2)	403 (10.0)	418 (10.3)
80-89 years	302 (3.7)	156 (3.9)	146 (3.6)
90-99 years	38 (0.5)	18 (0.4)	20 (0.5)
≥100 years	0 (0.0)	0 (0.0)	0 (0.0)
Indigenous self-identification	58 (0.7)	21 (0.5)	37 (0.9)
Occupation			
Technical services	133 (1.6)	63 (1.6)	70 (1.7)
Education	236 (2.9)	131 (3.2)	105 (2.6)
Healthcare	1114 (13.8)	459 (11.4)	655 (16.2)
Agricultural activities	15 (0.2)	10 (0.2)	5 (0.1)
Commerce	2532 (31.3)	1454 (36.0)	1078 (26.7)
Other	1618 (20.0)	707 (17.5)	911 (22.5)
Unemployed	483 (6.0)	206 (5.1)	277 (6.8)
Stay-at-home	1957 (24.2)	1014 (25.1)	943 (23.3)
Last-season flu vaccination	1396 (17.3)	701 (17.3)	695 (17.2)
Comorbidities			
Diabetes	1783 (22.0)	873 (21.6)	910 (22.5)
COPD	239 (3.0)	120 (3.0)	119 (2.9)
Asthma	181 (2.2)	85 (2.1)	96 (2.4)
Immunosuppression	145 (1.8)	61 (1.5)	84 (2.1)
Hypertension	2144 (26.5)	1070 (26.5)	1074 (26.6)
HIV/AIDS	56 (0.7)	26 (0.6)	30 (0.7)
Cardiovascular disease	276 (3.4)	123 (3.0)	153 (3.8)
Obesity	1716 (21.2)	879 (21.7)	837 (20.7)
Chronic kidney disease	266 (3.3)	116 (2.9)	150 (3.7)
Smoker	924 (11.4)	463 (11.4)	461 (11.4)
Type of medical attention			
Ambulatory	4794 (59.3)	2782 (68.8)	2012 (49.8)
Hospitalization	3294 (40.7)	1262 (31.2)	2032 (50.2)
Severity of the disease			
Non-critical	7232 (89.4)	3714 (91.8)	3518 (87.0)
Critical	856 (10.6)	330 (8.2)	526 (13.0)
Time from symptom onset to medical attention	4.7 (3.9)	4.5 (3.9)	4.9 (3.9)

	Total<br>n=8088	Antiviral<br>n=4044	Control<br>n=4044
Baseline symptoms			
Fever	6706 (82.9)	3374 (83.4)	3332 (82.4)
Cough	6646 (82.2)	3240 (80.1)	3406 (84.2)
Sore throat	3985 (49.3)	1971 (48.7)	2014 (49.8)
Shortness of breath	4448 (55.0)	2214 (54.7)	2234 (55.2)
Irritability	1947 (24.1)	868 (21.5)	1079 (26.7)
Diarrhea	2292 (28.3)	1140 (28.2)	1152 (28.5)
Chest pain	3122 (38.6)	1460 (36.1)	1662 (41.1)
Chills	4006 (49.5)	1868 (46.2)	2138 (52.9)
Headache	6422 (79.4)	3138 (77.6)	3284 (81.2)
Myalgias	5306 (65.6)	2673 (66.1)	2633 (65.1)
Arthralgias	4810 (59.5)	2433 (60.2)	2377 (58.8)
Abrupt deterioration	4995 (61.8)	2291 (56.7)	2704 (66.9)
Rhinorrhea	2599 (32.1)	1249 (30.9)	1350 (33.4)
Polypnea	1830 (22.6)	749 (18.5)	1081 (26.7)
Vomit	888 (11.0)	409 (10.1)	479 (11.8)
Abdominal pain	1693 (20.9)	668 (16.5)	1025 (25.3)
Conjunctivitis	1116 (13.8)	603 (14.9)	513 (12.7)
Cyanosis	719 (8.9)	256 (6.3)	463 (11.4)
Sudden onset of symptoms	3191 (39.5)	1574 (38.9)	1617 (40.0)

Data expressed as frequency (%) or mean (SD)

SD: Standard deviation, COPD: Chronic obstructive pulmonary disease, HIV/AIDS: Human immunodeficiency virus/acquired immune deficiency syndrome

EXCLI Journal 2021;20:199-222 – ISSN 1611-2156
Received: January 19, 2021, accepted: February 01, 2021, published: February 04, 2021

Supplementary Table 18: Baseline characteristics of patients with COVID-19 treated with Oseltamivir and propensity score-matched controls not treated with antivirals or antibiotics

	Total n=7574	Oseltamivir n=3787	Control n=3787
Sex			
Women	3351 (44.2)	1676 (44.3)	1675 (44.2)
Men	4223 (55.8)	2111 (55.7)	2112 (55.8)
Age, mean (SD)	50.8 (16.5)	50.8 (16.6)	50.7 (16.4)
Age categories			
0-19 years	98 (1.3)	47 (1.2)	51 (1.3)
20-29 years	698 (9.2)	349 (9.2)	349 (9.2)
30-39 years	1267 (16.7)	632 (16.7)	635 (16.8)
40-49 years	1569 (20.7)	788 (20.8)	781 (20.6)
50-59 years	1624 (21.4)	822 (21.7)	802 (21.2)
60-69 years	1213 (16.0)	607 (16.0)	606 (16.0)
70-79 years	785 (10.4)	386 (10.2)	399 (10.5)
80-89 years	282 (3.7)	138 (3.6)	144 (3.8)
90-99 years	38 (0.5)	18 (0.5)	20 (0.5)
≥100 years	0 (0.0)	0 (0.0)	0 (0.0)
Indigenous self-identification	51 (0.7)	17 (0.4)	34 (0.9)
Occupation			
Technical services	127 (1.7)	58 (1.5)	69 (1.8)
Education	202 (2.7)	112 (3.0)	90 (2.4)
Healthcare	1027 (13.6)	408 (10.8)	619 (16.3)
Agricultural activities	17 (0.2)	12 (0.3)	5 (0.1)
Commerce	2388 (31.5)	1394 (36.8)	994 (26.2)
Other	1484 (19.6)	626 (16.5)	858 (22.7)
Unemployed	464 (6.1)	204 (5.4)	260 (6.9)
Stay-at-home	1865 (24.6)	973 (25.7)	892 (23.6)
Last-season flu vaccination	1297 (17.1)	660 (17.4)	637 (16.8)
Comorbidities			
Diabetes	1730 (22.8)	853 (22.5)	877 (23.2)
COPD	219 (2.9)	102 (2.7)	117 (3.1)
Asthma	177 (2.3)	88 (2.3)	89 (2.4)
Immunosuppression	142 (1.9)	61 (1.6)	81 (2.1)
Hypertension	2070 (27.3)	1031 (27.2)	1039 (27.4)
HIV/AIDS	47 (0.6)	17 (0.4)	30 (0.8)
Cardiovascular disease	263 (3.5)	114 (3.0)	149 (3.9)
Obesity	1583 (20.9)	796 (21.0)	787 (20.8)
Chronic kidney disease	259 (3.4)	113 (3.0)	146 (3.9)
Smoker	864 (11.4)	436 (11.5)	428 (11.3)
Type of medical attention			
Ambulatory	4412 (58.3)	2588 (68.3)	1824 (48.2)
Hospitalization	3162 (41.7)	1199 (31.7)	1963 (51.8)
Severity of the disease			
Non-critical	6760 (89.3)	3474 (91.7)	3286 (86.8)
Critical	814 (10.7)	313 (8.3)	501 (13.2)
Time from symptom onset to medical attention	4.7 (3.9)	4.4 (3.9)	4.9 (4.0)

EXCLI Journal 2021;20:199-222 – ISSN 1611-2156
Received: January 19, 2021, accepted: February 01, 2021, published: February 04, 2021

	Total n=7574	Oseltamivir n=3787	Control n=3787
Baseline symptoms			
Fever	6370 (84.1)	3204 (84.6)	3166 (83.6)
Cough	6220 (82.1)	3013 (79.6)	3207 (84.7)
Sore throat	3719 (49.1)	1831 (48.3)	1888 (49.9)
Shortness of breath	4286 (56.6)	2137 (56.4)	2149 (56.7)
Irritability	1895 (25.0)	882 (23.3)	1013 (26.7)
Diarrhea	2127 (28.1)	1043 (27.5)	1084 (28.6)
Chest pain	2975 (39.3)	1388 (36.7)	1587 (41.9)
Chills	3768 (49.7)	1758 (46.4)	2010 (53.1)
Headache	6046 (79.8)	2947 (77.8)	3099 (81.8)
Myalgias	4970 (65.6)	2508 (66.2)	2462 (65.0)
Arthralgias	4506 (59.5)	2273 (60.0)	2233 (59.0)
Abrupt deterioration	4746 (62.7)	2187 (57.8)	2559 (67.6)
Rhinorrhea	2450 (32.3)	1198 (31.6)	1252 (33.1)
Polypnea	1834 (24.2)	790 (20.9)	1044 (27.6)
Vomit	853 (11.3)	395 (10.4)	458 (12.1)
Abdominal pain	1585 (20.9)	608 (16.1)	977 (25.8)
Conjunctivitis	1014 (13.4)	540 (14.3)	474 (12.5)
Cyanosis	745 (9.8)	291 (7.7)	454 (12.0)
Sudden onset of symptoms	3024 (39.9)	1494 (39.5)	1530 (40.4)

Data expressed as frequency (%) or mean (SD)

SD: Standard deviation, COPD: Chronic obstructive pulmonary disease, HIV/AIDS: Human immunodeficiency virus/acquired immune deficiency syndrome

EXCLI Journal 2021;20:199-222 – ISSN 1611-2156
Received: January 19, 2021, accepted: February 01, 2021, published: February 04, 2021

Supplementary Table 19: Baseline characteristics of patients with COVID-19 treated with antibiotics and propensity score-matched controls not treated with antivirals or antibiotics

	Total n=27486	Antibiotic n=13743	Control n=13743
Sex			
Women	11940 (43.4)	5940 (43.2)	6000 (43.7)
Men	15546 (56.6)	7803 (56.8)	7743 (56.3)
Age, mean (SD)	48.2 (16.7)	48.3 (16.8)	48.1 (16.6)
Age categories			
0-19 years	928 (3.4)	445 (3.2)	483 (3.5)
20-29 years	2778 (10.1)	1399 (10.2)	1379 (10.0)
30-39 years	4912 (17.9)	2475 (18.0)	2437 (17.7)
40-49 years	5976 (21.7)	3010 (21.9)	2966 (21.6)
50-59 years	5830 (21.2)	2924 (21.3)	2906 (21.1)
60-69 years	4104 (14.9)	2056 (15.0)	2048 (14.9)
70-79 years	2097 (7.6)	1025 (7.5)	1072 (7.8)
80-89 years	744 (2.7)	355 (2.6)	389 (2.8)
90-99 years	111 (0.4)	52 (0.4)	59 (0.4)
≥100 years	6 (0.0)	2 (0.0)	4 (0.0)
Indigenous self-identification	152 (0.6)	60 (0.4)	92 (0.7)
Occupation			
Technical services	466 (1.7)	229 (1.7)	237 (1.7)
Education	1284 (4.7)	622 (4.5)	662 (4.8)
Healthcare	2829 (10.3)	1555 (11.3)	1274 (9.3)
Agricultural activities	90 (0.3)	39 (0.3)	51 (0.4)
Commerce	10282 (37.4)	5227 (38.0)	5055 (36.8)
Other	4993 (18.2)	2394 (17.4)	2599 (18.9)
Unemployed	1131 (4.1)	674 (4.9)	457 (3.3)
Stay-at-home	6411 (23.3)	3003 (21.9)	3408 (24.8)
Last-season flu vaccination	5289 (19.2)	2538 (18.5)	2751 (20.0)
Comorbidities			
Diabetes	5268 (19.2)	2591 (18.9)	2677 (19.5)
COPD	480 (1.7)	232 (1.7)	248 (1.8)
Asthma	622 (2.3)	334 (2.4)	288 (2.1)
Immunosuppression	406 (1.5)	171 (1.2)	235 (1.7)
Hypertension	6049 (22.0)	2976 (21.7)	3073 (22.4)
HIV/AIDS	120 (0.4)	58 (0.4)	62 (0.5)
Cardiovascular disease	659 (2.4)	289 (2.1)	370 (2.7)
Obesity	6009 (21.9)	3026 (22.0)	2983 (21.7)
Chronic kidney disease	531 (1.9)	231 (1.7)	300 (2.2)
Smoker	3115 (11.3)	1554 (11.3)	1561 (11.4)
Type of medical attention			
Ambulatory	18548 (67.5)	10280 (74.8)	8268 (60.2)
Hospitalization	8938 (32.5)	3463 (25.2)	5475 (39.8)
Severity of the disease			
Non-critical	24844 (90.4)	12826 (93.3)	12018 (87.4)
Critical	2642 (9.6)	917 (6.7)	1725 (12.6)
Time from symptom onset to medical attention	5.1 (3.9)	5.5 (3.8)	4.8 (3.9)

	Total n=27486	Antibiotic n=13743	Control n=13743
Baseline symptoms			
Fever	19681 (71.6)	9912 (72.1)	9769 (71.1)
Cough	20783 (75.6)	10412 (75.8)	10371 (75.5)
Sore throat	12673 (46.1)	6432 (46.8)	6241 (45.4)
Shortness of breath	12972 (47.2)	6471 (47.1)	6501 (47.3)
Irritability	5405 (19.7)	2880 (21.0)	2525 (18.4)
Diarrhea	7083 (25.8)	3589 (26.1)	3494 (25.4)
Chest pain	8782 (32.0)	4613 (33.6)	4169 (30.3)
Chills	10668 (38.8)	5665 (41.2)	5003 (36.4)
Headache	19521 (71.0)	10121 (73.6)	9400 (68.4)
Myalgias	16206 (59.0)	8161 (59.4)	8045 (58.5)
Arthralgias	14959 (54.4)	7530 (54.8)	7429 (54.1)
Abrupt deterioration	14853 (54.0)	7071 (51.5)	7782 (56.6)
Rhinorrhea	7814 (28.4)	4126 (30.0)	3688 (26.8)
Polypnea	4169 (15.2)	2200 (16.0)	1969 (14.3)
Vomit	2353 (8.6)	1237 (9.0)	1116 (8.1)
Abdominal pain	3643 (13.3)	2034 (14.8)	1609 (11.7)
Conjunctivitis	3532 (12.9)	1913 (13.9)	1619 (11.8)
Cyanosis	1420 (5.2)	808 (5.9)	612 (4.5)
Sudden onset of symptoms	10253 (37.3)	5089 (37.0)	5164 (37.6)

Data expressed as frequency (%) or mean (SD)

SD: Standard deviation, COPD: Chronic obstructive pulmonary disease, HIV/AIDS: Human immunodeficiency virus/acquired immune deficiency syndrome

EXCLI Journal 2021;20:199-222 – ISSN 1611-2156
Received: January 19, 2021, accepted: February 01, 2021, published: February 04, 2021

Supplementary Table 20: Baseline characteristics of 903 patients with data of timing of initiation of antiviral treatment with laboratory-confirmed COVID-19, who received medical attention in one of 154 accredited medical units in Mexico City

	Total n=903	Acyclovir n=26	Amantadine n=131	Lopinavir-Ritonavir n=9	Oseltamivir n=702	Rimantadine n=24	Zanamivir n=11
Sex							
Women	403 (44.6)	14 (53.8)	72 (55)	2 (22.2)	294 (41.9)	13 (54.2)	8 (72.7)
Men	500 (55.4)	12 (46.2)	59 (45)	7 (77.8)	408 (58.1)	11 (45.8)	3 (27.3)
Age, mean (SD)	46.7 (14.8)	44.5 (13.9)	42.3 (14.3)	51 (11.1)	47.8 (14.8)	42.6 (13.9)	40 (13.9)
Age categories							
0-19 years	17 (1.9)	0 (0)	5 (3.8)	0 (0)	11 (1.6)	1 (4.2)	0 (0)
20-29 years	88 (9.7)	5 (19.2)	18 (13.7)	0 (0)	62 (8.8)	1 (4.2)	2 (18.2)
30-39 years	204 (22.6)	5 (19.2)	40 (30.5)	0 (0)	147 (20.9)	8 (33.3)	4 (36.4)
40-49 years	220 (24.4)	7 (26.9)	26 (19.8)	5 (55.6)	170 (24.2)	10 (41.7)	2 (18.2)
50-59 years	192(21.3)	4 (15.4)	25 (19.1)	2 (22.2)	157 (22.4)	2 (8.3)	2 (18.2)
60-69 years	118 (13.1)	5 (19.2)	11 (8.4)	1 (11.1)	100 (14.2)	0 (0)	1 (9.1)
70-79 years	51 (5.6)	0 (0)	5 (3.8)	1 (11.1)	43 (6.1)	2 (8.3)	0 (0)
80-89 years	10 (1.1)	0 (0)	1 (0.8)	0 (0)	9 (1.3)	0 (0)	0 (0)
90-99 years	3 (0.3)	0 (0)	0 (0)	0 (0)	3 (0.4)	0 (0)	0 (0)
Indigenous self-identification	10 (1.1)	0 (0)	1 (0.8)	0 (0)	7 (1)	0 (0)	2 (18.2)
Occupation							
Technical services	29 (3.2)	0 (0)	3 (2.3)	0 (0)	25 (3.6)	0 (0)	1 (9.1)
Education	34 (3.8)	0 (0)	8 (6.1)	0 (0)	25 (3.6)	1 (4.2)	0 (0)
Healthcare	117 (13)	2 (7.7)	12 (9.2)	2 (22.2)	97 (13.8)	4 (16.7)	0 (0)
Agricultural activities	2 (0.2)	0 (0)	0 (0)	0 (0.0)	2 (0.3)	0 (0)	0 (0)
Commerce	327 (36.2)	12 (46.2)	53 (40.5)	0 (0.0)	251 (35.8)	8 (33.3)	3 (27.3)
Other	197 (21.8)	5 (19.2)	31 (23.7)	6 (66.7)	149 (21.2)	5 (20.8)	1 (9.1)
Unemployed	17 (1.9)	0 (0)	0 (0)	1 (11.1)	15 (2.1)	1 (4.2)	0 (0)
Stay-at-home	180 19.9)	7 (26.9)	24 (18.3)	0 (0)	138 (19.7)	5 (20.8)	6 (54.5)
Last-season flu vaccination	222 (24.6)	5 (19.2)	38 (29)	1 (11.1)	173 (24.6)	4 (16.7)	1 (9.1)
Special populations							
Children and adolescents (<18 years)	11 (1.2)	0 (0.0)	3 (2.3)	0 (0)	7 (1)	1 (4.2)	0 (0)
Age, mean (SD)	11.6 (5.5)	-	11 (7.8)	-	11.9 (5.5)	12	-
Last-season flu vaccination	2 (18.2)	0 (0)	0 (0)	0 (0)	2 (28.6)	0 (0)	0 (0)
Non-pregnant/puerperal adults (≥18 years)	890 (98.6)	26 (100)	128 (97.7)	9 (100)	693 (98.7)	23 (95.8)	11 (100)
Age, mean (SD)	47.2 (14.3)	44.5 (13.9)	42.9 (13.6)	51 (11.1)	48.2 (14.2)	43.9 (12.6)	40 (12.9)
Last-season flu vaccination	220 (24.7)	5 (19.2)	38 (29.7)	1 (11.1)	171 (24.7)	4 (17.4)	1 (9.1)

EXCLI Journal 2021;20:199-222 – ISSN 1611-2156
Received: January 19, 2021, accepted: February 01, 2021, published: February 04, 2021

	Total n=903	Acyclovir n=26	Amantadine n=131	Lopinavir-Ritonavir n=9	Oseltamivir n=702	Rimantadine n=24	Zanamivir n=11
Comorbidities							
Diabetes	138 (15.3)	0 (0)	10 (7.6)	2 (22.2)	123 (17.5)	3 (12.5)	0 (0)
COPD	12 (1.3)	0 (0)	2 (1.5)	0 (0)	10 (1.4)	0 (0)	0 (0)
Asthma	23 (2.5)	1 (3.8)	3 (2.3)	1 (11.1)	18 (2.6)	0 (0)	0 (0)
Immunosuppression	14 (1.6)	0 (0)	5 (3.8)	0 (0)	9 (1.3)	0 (0)	0 (0)
Hypertension	166 (18.4)	4 (15.4)	11 (8.4)	0 (0)	147 (20.9)	3 (12.5)	1 (9.1)
HIV/AIDS	2 (0.2)	0 (0)	0 (0)	0 (0)	2 (0.3)	0 (0)	0 (0)
Cardiovascular disease	27 (1.6)	2 (7.7)	1 (0.8)	1 (11.1)	23 (3.3)	0 (0)	0 (0)
Obesity	241 (3)	9 (34.6)	41 (31.3)	2 (22.2)	182 (25.9)	3 (12.5)	4 (36.4)
Chronic kidney disease	17 (26.7)	0 (0)	0 (0)	0 (0)	17 (2.4)	0 (0)	0 (0)
Smoker	107 (1.9)	0 (0)	20 (15.3)	1 (11.1)	82 (11.7)	2 (8.3)	2 (18.2)
Type of medical attention							
Ambulatory	675 (74.8)	26 (100)	127 (96.9)	0 (0)	491 (69.9)	22 (91.7)	9 (81.8)
Hospitalization	228 (25.2)	0 (0)	4 (3.1)	9 (10)	211 (30.1)	2 (8.3)	2 (18.2)
Severity of the disease							
Non-critical	833 (92.2)	26 (100)	130 (99.2)	4 (44.4)	638 (90.9)	24 (100)	11 (100)
Critical	70 (7.8)	0 (0)	1 (0.8)	5 (55.6)	64 (9.1)	0 (0)	0 (0)
Baseline symptoms							
Fever	643 (71.2)	20 (76.9)	82 (62.6)	9 (100)	511 (72.8)	12 (50)	9 (81.8)
Cough	675 (74.8)	17 (65.4)	98 (74.8)	7 (77.8)	527 (75.1)	17 (70.8)	9 (81.8)
Sore throat	434 (48.1)	10 (38.5)	63 (48.1)	3 (33.3)	338 (48.1)	14 (58.3)	6 (54.5)
Shortness of breath	402 (44.5)	11 (42.3)	36 (27.5)	6 (66.7)	335 (47.7)	9 (37.5)	5 (45.5)
Irritability	199 (22)	5 (19.2)	23 (17.6)	1 (11.1)	158 (22.5)	6 (25)	6 (54.5)
Diarrhea	285 (31.6)	9 (34.6)	39 (29.8)	1 (11.1)	226 (32.2)	4 (16.7)	6 (54.5)
Chest pain	330 (36.5)	8 (30.8)	37 (28.2)	3 (33.3)	266 (37.9)	9 (37.5)	7 (63.6)
Chills	434 (48.1)	12 (46.2)	60 (45.8)	3 (33.3)	342 (48.7)	11 (45.8)	6 (54.5)
Headache	645 (71.4)	19 (73.1)	93 (71)	7 (77.8)	502 (71.5)	15 (62.5)	9 (81.8)
Myalgias	590 (65.3)	20 (76.9)	78 (59.5)	5 (55.6)	464 (66.1)	15 (62.5)	8 (72.7)
Arthralgias	552 (61.1)	16 (61.5)	73 (55.7)	5 (55.6)	436 (62.1)	16 (66.7)	6 (54.5)
Abrupt deterioration	556 (61.6)	15 (57.7)	77 (58.8)	6 (66.7)	440 (62.7)	12 (50)	6 (54.5)
Rhinorrhea	294 (32.6)	5 (19.2)	45 (34.4)	1 (11.1)	229 (32.6)	10 (41.7)	4 (36.4)
Polypnea	171 (18.9)	3 (11.5)	22 (16.8)	1 (11.1)	141 (20.1)	2 (8.3)	2 (18.2)
Vomit	106 (11.7)	2 (7.7)	9 (6.9)	1 (11.1)	90 (12.8)	3 (12.5)	1 (9.1)
Abdominal pain	153 (16.9)	1 (3.8)	17 (13)	2 (22.2)	127 (18.1)	3 (12.5)	3 (27.3)
Conjunctivitis	157 (17.4)	4 (15.4)	20 (15.3)	1 (11.1)	123 (17.5)	5 (20.8)	4 (36.4)

EXCLI Journal 2021;20:199-222 – ISSN 1611-2156
Received: January 19, 2021, accepted: February 01, 2021, published: February 04, 2021

	Total n=903	Acyclovir n=26	Amantadine n=131	Lopinavir-Ritonavir n=9	Oseltamivir n=702	Rimantadine n=24	Zanamivir n=11
Cyanosis	67 (7.4)	1 (3.8)	7 (5.3)	1 (11.1)	56 (8)	1 (4.2)	1 (9.1)
Sudden onset of symptoms	349 (38.6)	7 (26.9)	47 (35.9)	5 (55.6)	274 (3)	12 (50)	4 (36.4)
Concomitant use of antibiotics	566 (62.7)	22 (84.6)	84 (64.1)	5 (55.6)	434 (61.8)	15 (62.5)	6 (54.5)
Timing of antiviral treatment							
Before medical attention	783 (86.7)	19 (73.1)	121 (92.4)	7 (77.8)	603 (85.9)	23 (95.8)	10 (90.9)
After medical attention	120 (13.3)	7 (26.9)	10 (7.6)	2 (22.2)	99 (14.1)	1 (4.2)	1 (9.1)
≤2 days after symptom onset	579 (64.2)	8 (30.8)	99 (75.6)	1 (11.1)	448 (63.9)	16 (66.7)	7 (63.6)
>2 days after symptom onset	323 (35.8)	18 (69.2)	32 (24.4)	8 (88.9)	253 (36.1)	8 (33.3)	4 (36.4)

Data expressed as frequency (%) or mean (SD)

SD: Standard deviation, COPD: Chronic obstructive pulmonary disease, HIV/AIDS: Human immunodeficiency virus/acquired immune deficiency syndrome

EXCLI Journal 2021;20:199-222 – ISSN 1611-2156
Received: January 19, 2021, accepted: February 01, 2021, published: February 04, 2021

Supplementary Table 21: Survival analysis of laboratory-confirmed COVID-19 patients according to early or late use of antivirals

Group	Total	Deaths	Survivors	Survival	p value
Early use of antivirals (≤2 days)	579	64	515	88.9%	-
Type of antiviral					
No antiviral/antibiotic	114143	7351	106792	93.6%	Reference
Acyclovir	8	0	8	100%	0.5
Amantadine	99	2	97	98%	0.08
Lopinavir-Ritonavir	1	0	1	100%	0.8
Oseltamivir	448	62	386	86.2%	<0.0001
Rimantadine	16	0	16	100%	0.3
Zanamivir	7	0	7	100%	0.5
Late use of antivirals (>2 days)	323	28	295	91.3%	-
Type of antiviral					
No antiviral/antibiotic	114143	7351	106792	93.6%	Reference
Acyclovir	18	0	18	100%	0.3
Amantadine	32	1	31	96.9%	0.4
Lopinavir-Ritonavir	8	2	6	75%	0.03
Oseltamivir	253	15	228	90.1%	0.04
Rimantadine	8	0	8	100%	0.5
Zanamivir	4	0	4	100%	0.6

Data expressed as number of patients or percentual of survival. p values were calculated by Log-Rank test.

EXCLI Journal 2021;20:199-222 – ISSN 1611-2156
Received: January 19, 2021, accepted: February 01, 2021, published: February 04, 2021

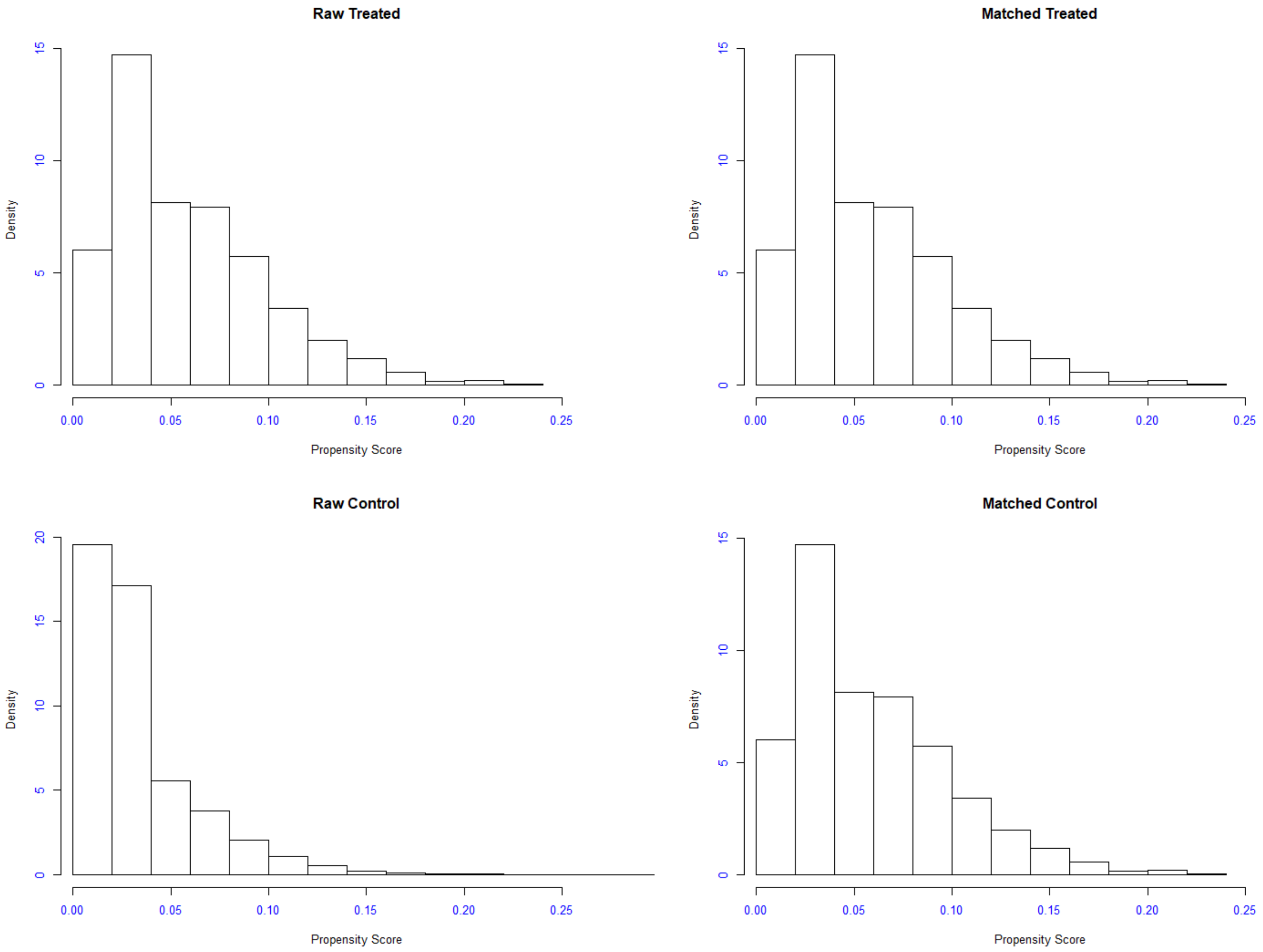

Supplementary Figure 1: Density functions of propensity score-matched patients treated with antivirals and controls before and after matching

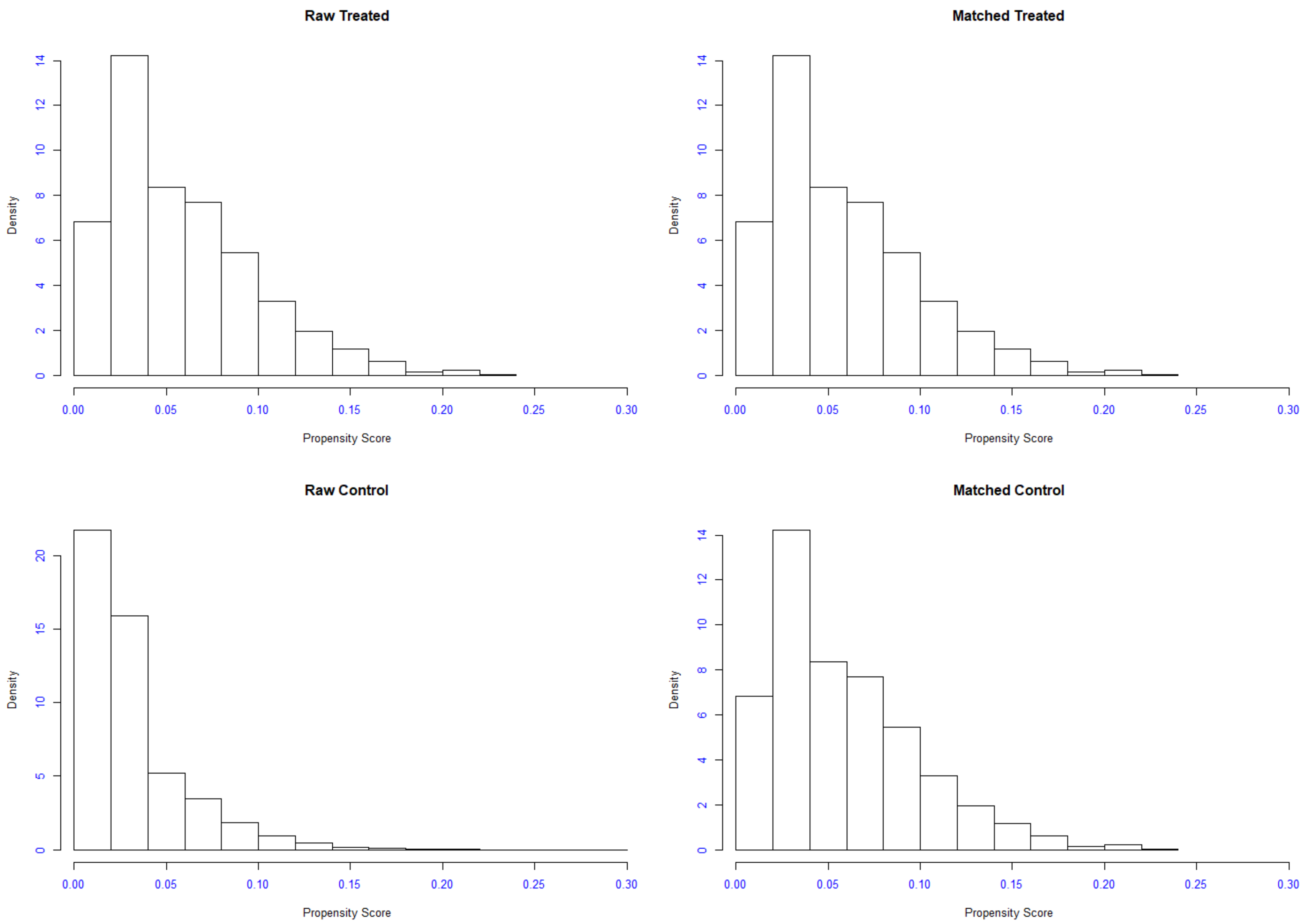

Supplementary Figure 2: Density functions of propensity score-matched patients treated with Oseltamivir and controls before and after matching

Received: January 19, 2021, accepted: February 01, 2021, published: February 04, 2021

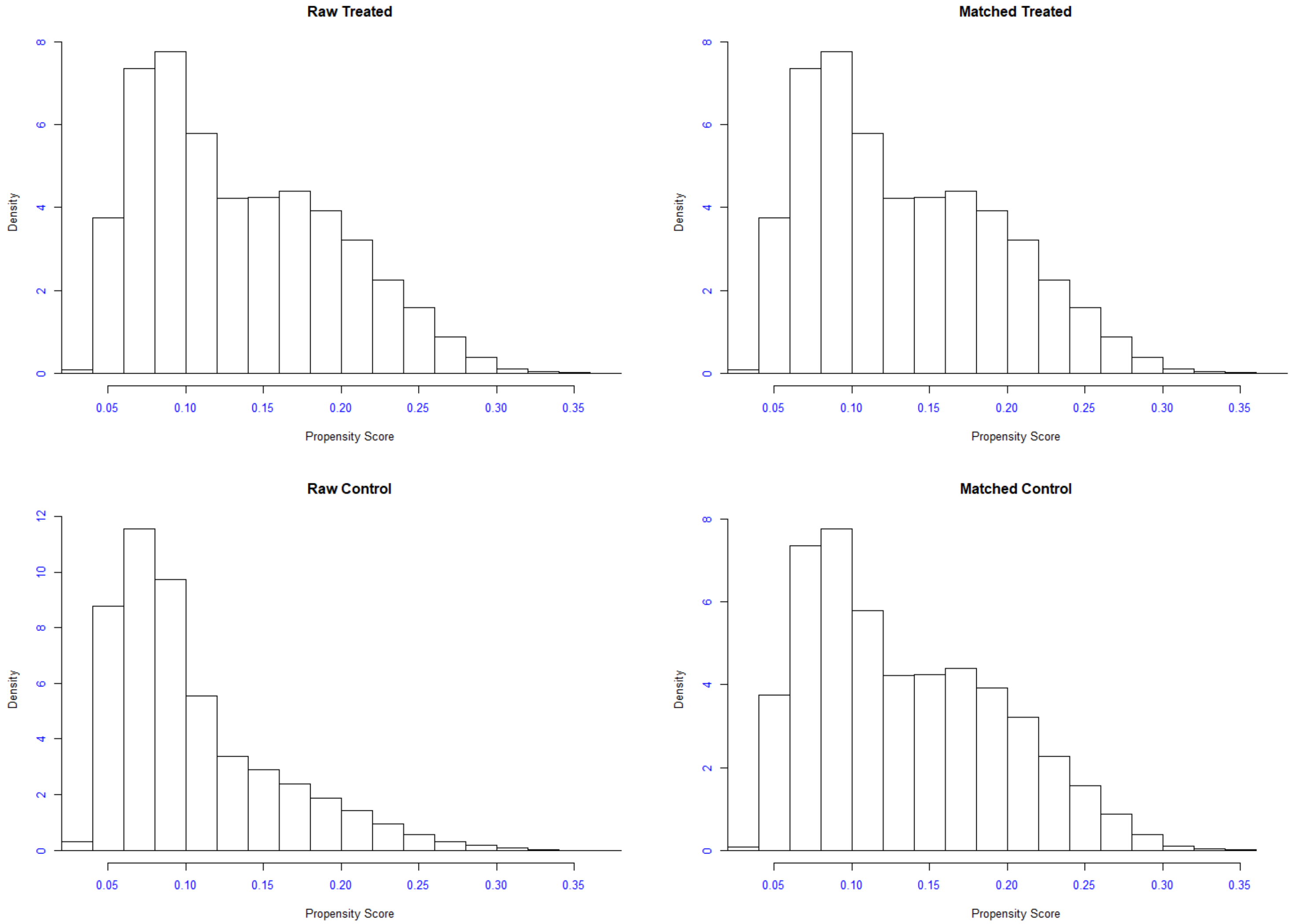

Supplementary Figure 3: Density functions of propensity score-matched patients treated with antibiotics and controls before and after matching

EXCLI Journal 2021;20:199-222 – ISSN 1611-2156
Received: January 19, 2021, accepted: February 01, 2021, published: February 04, 2021

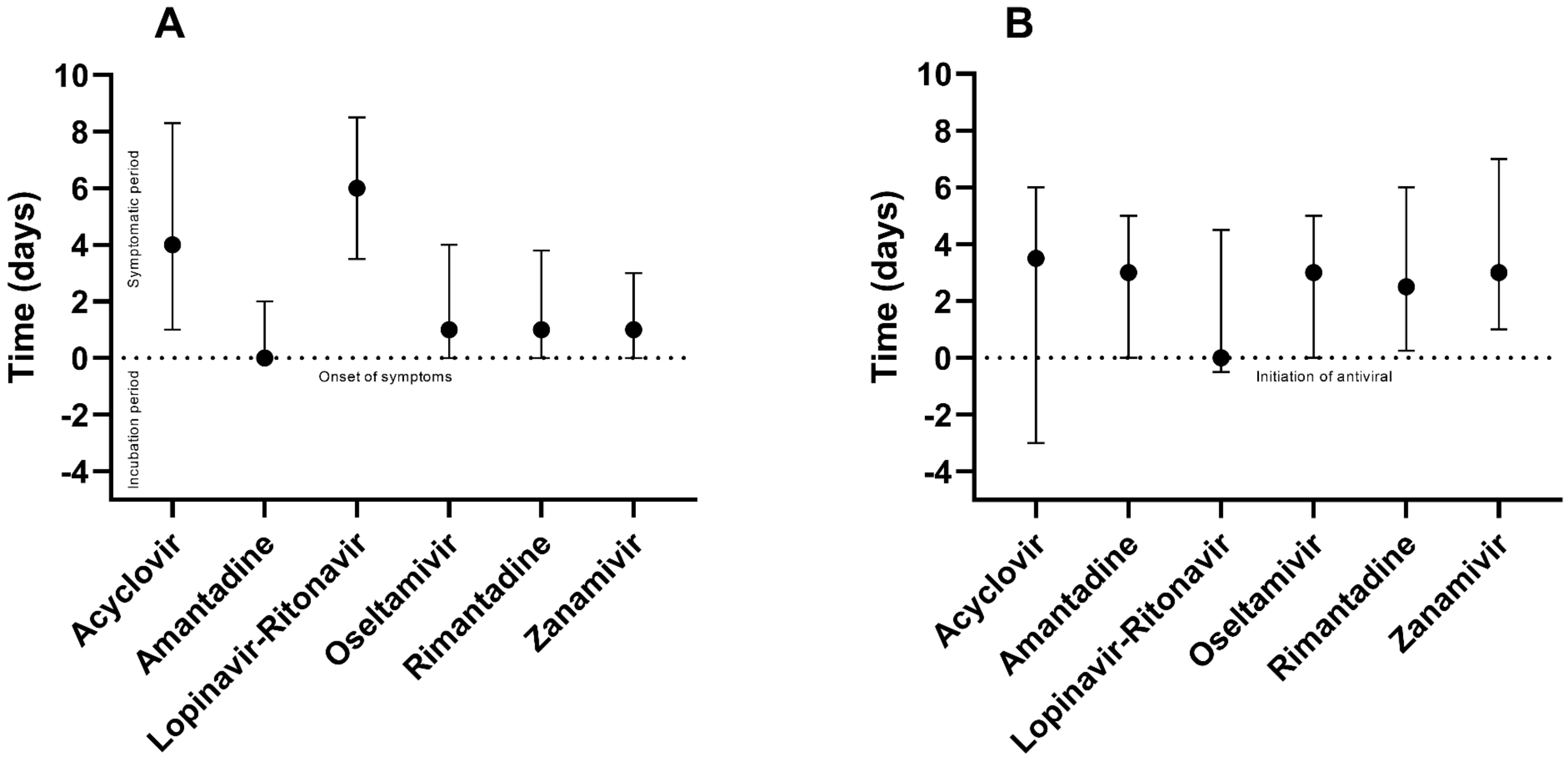

Supplementary Figure 4: Time from symptom onset to initiation of antivirals (**A**) and time from initiation of antivirals to hospitalization (**B**) in 903 patients with laboratory-confirmed COVID-19 receiving medical attention in one of 154 accredited medical units in Mexico City

EXCLI Journal 2021;20:199-222 – ISSN 1611-2156
Received: January 19, 2021, accepted: February 01, 2021, published: February 04, 2021

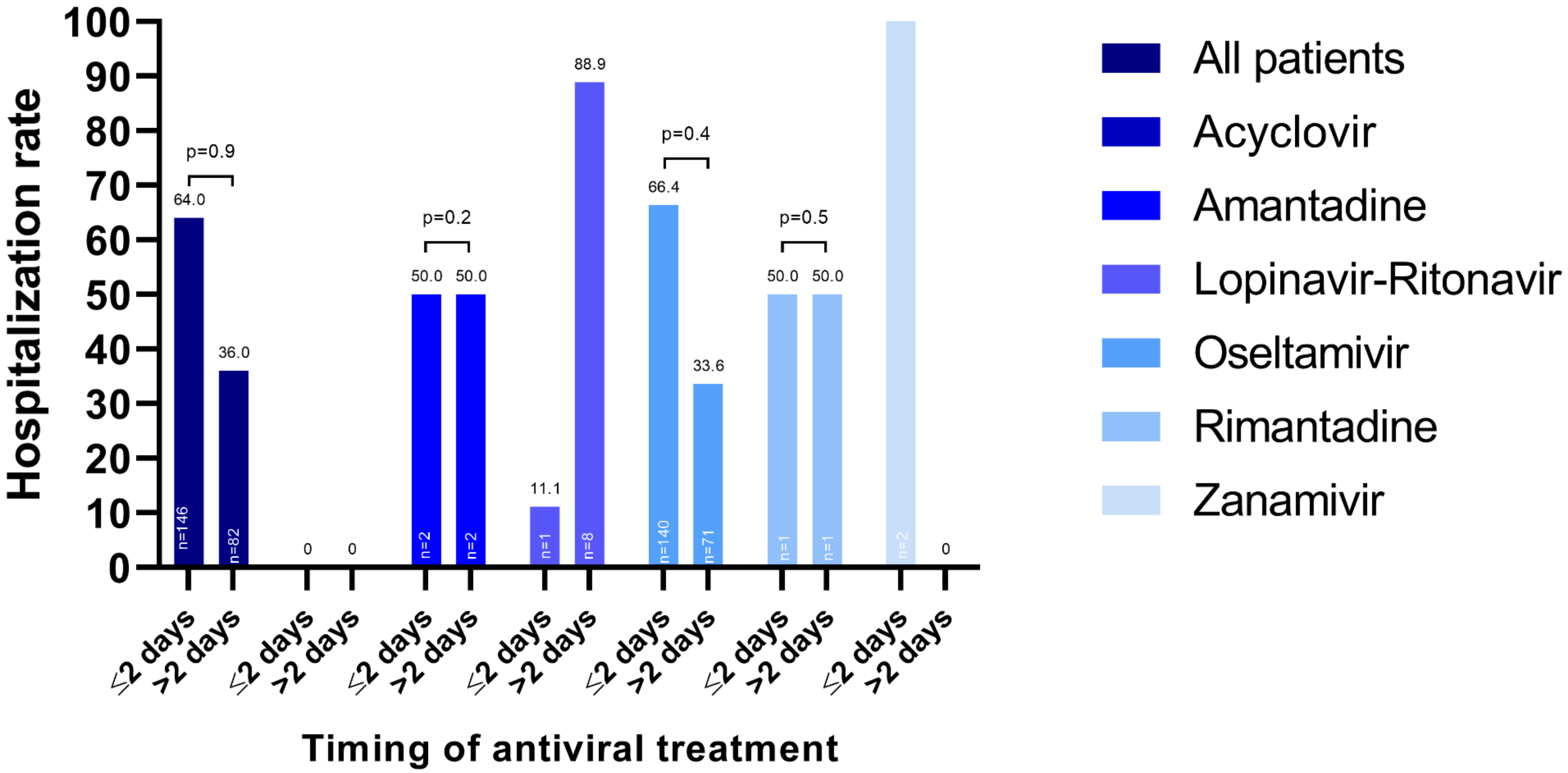

Supplementary Figure 5: Comparisons of hospitalization rates in 903 patients with laboratory-confirmed COVID-19 receiving medical attention in one of 154 accredited medical units in Mexico City who received early (≤2 days) or late (>2 days) antivirals
